# Arabidopsis phospholipase Dα1 and Dδ oppositely modulate EDS1- and SA-independent basal resistance against adapted powdery mildew

**DOI:** 10.1093/jxb/ery146

**Published:** 2018-04-18

**Authors:** Qiong Zhang, Robert Berkey, Joshua J Blakeslee, Jinshan Lin, Xianfeng Ma, Harlan King, Anna Liddle, Liang Guo, Teun Munnik, Xuemin Wang, Shunyuan Xiao

**Affiliations:** 1 Institute for Bioscience and Biotechnology Research, University of Maryland, Rockville, MD, USA; 2 Department of Horticulture and Crop Science, Ohio Agricultural Research and Development Center, The Ohio State University, Wooster, OH, USA; 3 National Key Laboratory of Crop Genetic Improvement, College of Plant Sciences, Huazhong Agricultural University, Wuhan, China; 4 Section of Plant Physiology, Swammerdam Institute for Life Sciences, University of Amsterdam, The Netherlands; 5 Department of Biology, University of Missouri, St. Louis, MO, USA; 6 Donald Danforth Plant Science Center, St. Louis, MO, USA; 7 Department of Plant Sciences and Landscape Architecture, University of Maryland, Rockville, MD, USA

**Keywords:** *Arabidopsis thaliana*, EDS1, *Hyaloperonospora arabidopsidis*, jasmonic acid, phospholipase D, plant defense signaling, post-penetration resistance, powdery mildew, salicylic acid

## Abstract

Plants use a tightly regulated immune system to fight off various pathogens. Phospholipase D (PLD) and its product, phosphatidic acid, have been shown to influence plant immunity; however, the underlying mechanisms remain unclear. Here, we show that the Arabidopsis mutants *pldα1* and *pldδ*, respectively, exhibited enhanced resistance and enhanced susceptibility to both well-adapted and poorly adapted powdery mildew pathogens, and a virulent oomycete pathogen, indicating that *PLDα1* negatively while *PLDδ* positively modulates post-penetration resistance. The *pldα1δ* double mutant showed a similar infection phenotype to *pldα1*, genetically placing *PLDα1* downstream of *PLDδ*. Detailed genetic analyses of *pldδ* with mutations in genes for salicylic acid (SA) synthesis (*SID2*) and/or signaling (*EDS1* and *PAD4*), measurement of SA and jasmonic acid (JA) levels, and expression of their respective reporter genes indicate that *PLDδ* contributes to basal resistance independent of EDS1/PAD4, SA, and JA

signaling. Interestingly, while PLDα1–enhanced green fluorescent protein (eGFP) was mainly found in the tonoplast before and after haustorium invasion, PLDδ–eGFP’s focal accumulation to the plasma membrane around the fungal penetration site appeared to be suppressed by adapted powdery mildew. Together, our results demonstrate that PLDα1 and PLDδ oppositely modulate basal, post-penetration resistance against powdery mildew through a non-canonical mechanism that is independent of EDS1/PAD4, SA, and JA.

## Teaser

*Phospholipase Dδ* (*PLDδ*) is involved in promoting basal, post-penetration resistance against powdery mildew through a potentially novel pathway(s) independent of EDS1/PAD4, SA, and JA, whereas *PLDα1* negatively modulates this process.

## Introduction

Many fungal and oomycete pathogens penetrate the plant cell wall and extract nutrients from host cells by a similar feeding structure called the haustorium. Plant defense against haustorium-forming pathogens exhibits clear spatiotemporal characteristics that can be conveniently divided into two distinct layers: penetration resistance (cell wall-based; the first layer) and post-penetration resistance (haustorium-targeted; the second layer). Penetration resistance is usually sufficient to stop non-adapted pathogens from entering the host cell by forming a papilla, which is cell wall thickening with deposition of callose (1,3-β-glucan) and other defense chemicals at the penetration site. This process is contributed by at least two independent mechanisms in Arabidopsis. One involves focal exocytosis of antimicrobial materials mediated by PENETRATION1 (PEN1), a syntaxin, and its SNARE partners (Collins *et al.*, 2003; Kwon *et al.*, 2008); the other engages the production of glucosinolates by PEN2 myrosinase and subsequent transport of such antifungal chemicals by the PEN3 ATP-binding cassette transporter (Lipka *et al.*, 2005; Stein *et al.*, 2006; Bednarek *et al.*, 2009). Both mechanisms are probably activated upon recognition of conserved pathogen-associated molecular patterns (PAMPs) by cell surface pattern recognition receptors (PRRs), and thus may be part of PAMP-triggered immunity (PTI) (Jones and Dangl, 2006; Hückelhoven and Panstruga, 2011).

Adapted fungi or oomycetes that can overcome penetration resistance face the second layer of plant defense. Despite successful penetration, early stage haustorial development and/or function can be inhibited by stage I post-penetration resistance which may continue to engage PTI and other defense mechanisms. However, once stage I post-penetration resistance is suppressed by effector proteins secreted from better-adapted pathogens, haustoria can establish function, and disease ensues. Plants have evolved stage II post-penetration resistance to defeat these better adapted pathogens through the action of plant resistance (R) proteins. Most characterized R proteins are intracellular immune receptors belonging to the nucleotide-binding site–leucine-rich repeat (NB-LRR) superfamily that detects the presence or activity of specific effector proteins termed avirulence factors (Avrs). Thus, stage II post-penetration resistance in many cases is equivalent to effector-triggered immunity (ETI), which often exhibits race specificity and features with rapid cell death at the infection site, namely the hypersensitive response (HR) (Jones and Dangl, 2006). Based on the N-terminal domains, NB-LRRs are divided into two major classes, Toll-interleukin 1 receptor (TIR)-NB-LRRs and coiled-coil (CC)-NB-LRRs. While characterized TIR-NB-LRRs require the nucleocytoplasmic lipase-like protein ENHANCED DISEASE SUSCEPTIBILITY 1 (EDS1) for signal transduction, most CC-NB-LRRs engage the plasma membrane (PM)-anchored integrin-like protein NON-RACE-SPECIFIC DISEASE RESISTANCE 1 (NDR1) for signaling (Cui *et al.*, 2015).

Detection of pathogens triggers a conserved signaling network regulated by salicylic acid (SA), jasmonic acid (JA), and ethylene (ET), resulting in the activation of defense responses including pathogenesis-related (*PR*) gene expression, reactive oxygen species (ROS) production, and callose deposition (Bari and Jones, 2009; Pieterse *et al.*, 2012). SA signaling plays a critical role in activation of local as well as systemic acquired resistance (SAR) to fight against biotrophic and hemi-biotrophic pathogens. Depending on the context of specific plant–pathogen interactions, the SA pathway could act antagonistically or synergistically with the JA/ET pathways, which are mainly effective against necrotrophic pathogens (Glazebrook, 2005; Robert-Seilaniantz *et al.*, 2011). EDS1 and its interacting homologous partner PHYTOALEXIN-DEFICIENT 4 (PAD4) are both required for adequate SA synthesis and signaling, and play a role in the antagonism between SA- and JA/ET-dependent defense pathways (Zhou *et al.*, 1998; Falk *et al.*, 1999; Feys *et al.*, 2001; Wiermer *et al.*, 2005). Furthermore, EDS1 and PAD4 have also been shown to regulate SA-independent defense responses (Feys *et al.*, 2005; Venugopal *et al.*, 2009; Zhu *et al.*, 2011; Wagner *et al.*, 2013; Cui *et al.*, 2017).

Two non-NB-LRR Arabidopsis R proteins, RPW8.1 and RPW8.2, confer broad-spectrum resistance to powdery mildew fungi (Xiao *et al.*, 2001), which requires EDS1, PAD4, and SA signaling (Xiao *et al.*, 2003; Xiao *et al.*, 2005). RPW8.2 is specifically targeted to the host-derived extra-haustorial membrane (EHM) encasing the fungal haustorium to activate on-site defenses including the formation of callose-enriched haustorial encasement and interface-focused H_2_O_2_ production to constrain the haustorium (Wang *et al.*, 2009; Berkey *et al.*, 2017). Previous studies suggest that a specific protein trafficking pathway is engaged for targeting RPW8.2 to the EHM (Wang *et al.*, 2013; Zhang *et al.*, 2015). However, how RPW8.2 achieves haustorium-targeted defense remains to be determined. A tempting speculation is that RPW8.2 may interact with a signaling lipid(s) to realize its specific targeting. In an effort to test this speculation, we instead found that two phospholipase D (PLD) enzymes play opposing roles in plant defense against powdery mildew fungi, but neither of them seems to be involved in RPW8-mediated resistance

PLD and its product phosphatidic acid (PA) have been implicated in modulating plant immunity. Exogenous SA treatment could induce higher PA levels as a result of PLD activity (Kalachova *et al.*, 2013; Rodas-Junco *et al.*, 2015), suggesting a positive role for PLD-derived PA; however, a limited number of genetic studies on *PLD* genes suggest that the outcome varies depending on the PLD isoforms involved and/or pathosystems examined. This is not surprising since there are 12 identified PLD isoforms [PLDα (3), PLDβ (2), PLDγ (3), PLDδ (1), PLDε (1), and PLDζ (2)] in Arabidopsis (Zhao, 2015; Zhang and Xiao, 2015; Hong *et al.* 2016). For example, Zhao *et al*. showed that genetic depletion of *PLDβ1* led to elevated levels of SA, ROS, SA-inducible gene expression, and enhanced resistance to the virulent bacterial strain *Pseudomonas syringae* tomato DC3000, indicating a negative role for *PLDβ1* in the SA signaling pathway (Zhao *et al.*, 2013). In contrast, Pinosa *et al*. reported that loss of *PLDδ* in Arabidopsis resulted in a higher penetration rate from two non-adapted powdery mildew fungi, barley mildew *Blumeria graminis* f.sp. *hordei* (*Bgh*) and pea mildew *Erysiphe pisi*, suggesting a positive role for *PLDδ* in penetration resistance (Pinosa *et al.*, 2013). However, despite the fact that repression of PLD-produced PA by *n*-butanol in Arabidopsis strongly inhibited the HR during ETI, not a single *PLD* gene was found to be responsible for this (Johansson *et al.*, 2014). Together, these studies suggest that PLDs play important roles in plant defenses with functional redundancy among family members. However, whether and how PLDs (or PLD-derived PA)-mediated signaling intersects with the well-defined SA and/or JA/ET signaling pathways is poorly understood (Zhao, 2015; Zhang and Xiao, 2015; Hong *et al.*, 2016).

In this study, we screened a panel of Arabidopsis mutants with T-DNA insertions in *PLD*, *pPLA* (*patatin-related phospholipase*), *PLC* (*phospholipase C*), *DGK* (*diacylglycerol kinase*), and *PIP5K* (*phosphatidylinositol 4-phosphate 5-kinase*) genes for an altered infection phenotype to adapted powdery mildew fungi. We found that while *PLDδ* knockout plants showed enhanced susceptibility, *PLDα1* knockout plants displayed enhanced resistance, suggesting that *PLDδ* and *PLDα1* play opposing roles in post-penetration resistance against powdery mildew. We thus conducted a detailed analysis to determine the genetic relationships between these two *PLD* genes, their possible involvement in PRW8.2’s localization and function, and the defense pathways they might modulate.

## Materials and methods

### Plant lines and growth conditions

All mutants used in this study were in the *Arabidopsis thaliana* accession Col-0 background. Sequence data of the genes in this article can be found in the Arabidopsis Genome Initiative or GenBank/EMBL databases. The accession numbers of all genes used in this study are listed in Supplementary Table S1 at *JXB* online. Mutants *sid2-2* (Wildermuth *et al.*, 2001), *eds1-2* (Bartsch *et al.*, 2006), *pad4-1* (Jirage *et al.*, 1999), *dde2-2* (von Malek *et al.*, 2002), *coi1-1* (Xie *et al.*, 1998), *pad4-1sid2-2* (Tsuda *et al.*, 2009), and *eds1-2pad4-1* (Kim *et al.*, 2014) have been described previously. The phospholipase-related mutants used for infection tests with *Golovinomyces cichoracearum* (*Gc*) UCSC1 are listed in Supplementary Table S1. The homozygous double (*sid2-2pldα1*, *eds1-2pldα1*, *pad4-1pldα1*, *sid2-2pldδ*, *eds1-2pldδ*, and *pad4-1pldδ*), triple (*pad4-1sid2-2pldα1*, *pad4-1sid2-2pldδ*, *eds1-2pad4-1pldδ*, and *eds1-2pad4-1sid2-2*), and quadruple (*eds1-2pad4-1sid2-2pldδ*) mutants were generated by genetic crosses and identified by PCR genotyping. S5/*pldα1* and S5/*pldδ* homozygous plants were made by crossing *pldα1* and *pldδ* to S5 (Xiao *et al.*, 2005) and subsequent PCR genotyping. All genotyping primers are listed in Supplementary Table S2.

Seeds were sown in Metro Mix 360 (Maryland Plant and Suppliers) and cold treated (4 °C for 2 d), and seedlings were grown under 22 °C, 65% relative humidity, short day (8 h light at 125 µmol m^−2^ s^−1^, 16 h dark).

### DNA constructs, plant transformation, and microscopy

For genetic complementation, the genomic sequences of *PLDα1* and *PLDδ* were amplified by PLDα1-F/PLDα1-R2 and PLDδ-F/PLDδ-R primers (Supplementary Table S2), respectively, using Q5 DNA polymerase (New England Biolabs, M0491L), cloned into pCX-SN (Chen *et al.*, 2009) containing the *35S* promoter, and introduced into *pldα1* and *pldδ*, respectively, via *Agrobacterium*-mediated transformation using the *A. tumefaciens* strain GV3101 (Clough and Bent, 1998).

For determining subcellular localizations of PLDα1 and PLDδ, the *p35S-pPLDα1:PLDα1-eGFP* (a 2 kb *PLDα1* untranslated promoter region and genomic sequence is amplified by the PLDα1-pF/PLDα1-R1 primer pairs), *p35S:PLDδ*- enhanced green fluorescent protein (*eGFP*), and *pPLDδ:PLDδ-eGFP* fusion constructs were made according to a previous report (Pinosa *et al.*, 2013). *p35S-pPLDα1:PLDα1-eGFP* was introduced into *pldα1* and Col-0, while *p35S:PLDδ-eGFP* and *pPLDδ:PLDδ-eGFP* were introduced into both *pldδ* and Col-0 via *Agrobacterium*-mediated transformation (Clough and Bent, 1998).

The expression and localization of the PLDα1–eGFP and PLDδ–eGFP fusion proteins were examined by confocal microscopy using a Zeiss LSM710 microscope (Wang *et al.*, 2013). Confocal images were processed using the ZEN software (2009 edition) from Carl Zeiss (http://www.well.ox.ac.uk/_asset/file/zeiss-elyra-quick-start-guide-pdf-2.pdf; last accessed 24 April 2018) and Adobe Photoshop CC.

### Pathogen infection, disease phenotyping, and quantification

Isolate *Gc* UCSC1 was maintained on Col-*nahG* plants, *Gc* UMSG1 on sow thistle plants (Wen *et al.*, 2011), and *Gc* UMSG3, a new isolate purified in the Xiao lab, on tobacco plants for fresh inocula. Inoculation, visual scoring of disease reaction phenotypes, and conidiophore quantification were done as previously described (Xiao *et al.*, 2005). Briefly, for conidiophore quantification, ~6 leaves per genotype were collected from sparsely and evenly inoculated 6-week-old plants at 4 days post-inoculation (dpi), cleared in a clearing solution (ethanol:phenol:acetic acid:glycerol=8:1:1:1, v/v/v/v), and stained by trypan blue solution (250 μg ml^−1^ in lactic acid:glycerol:water=1:1:1, v/v/v) for visualizing the fungal structure under the microscope. For each experiment, the total number of conidiophores per fungal colony was counted for at least 20 colonies per genotype. Data combined from three independent experiments were presented in a boxplot. For spore quantification, 4–6 leaf samples (~150 mg leaves per sample) per genotype from 6- to 7-week-old plants at 10–13 dpi were collected. A spore suspension of each sample was made by vortexing the leaves for 1 min in 40 ml of H_2_O (0.02% Silwet L-77) and used (diluted if necessary for susceptible genotypes) for spore counting using a hemocytometer under a dissecting microscope. Spore counts were normalized to the fresh weight of the corresponding leaf samples. All data analyses were done in R (R Core Team, 2014), and graphics were generated using ‘ggplot2’ (Wickham, 2009).

Assays with oomycete strains *Hyaloperonospora arabidopsidis* Noco2 and Emwa1, and bacterial strains *Pseudomonas syringae* pv. *maculicola* (*Pma*) ES4326, *Pma avrRpm1*, *Pma avrRps4*, and *Pma ∆hrcC* were done according to previous reports (Bonardi *et al.*, 2011; Tornero and Dangl, 2001).

### *In situ* detection of H_2_O_2_ accumulation and callose deposition

*In situ* H_2_O_2_ production and accumulation in the haustorium-invaded epidermal cells were stained and assessed using DAB (3,3'-diaminobenzidine) solution (Thordal-Christensen *et al.*, 1997). Callose deposition at the fungal penetration sites and around the haustorium was detected and evaluated by aniline blue staining. Light microscopy images were viewed using Zeiss Imager A1.

### Determination of endogenous SA, JA, and ABA concentrations

Three leaf samples of 6- to 7-week-old plants (~150 mg per sample) per genotype were harvested before and at 5 dpi with *Gc* UCSC1 for determining endogenous SA, JA, and abscisic acid (ABA) concentrations simultaneously. Phytohormone analyses were done as described previously for auxins (Novák *et al.*, 2012; Blakeslee and Murphy, 2016), with the following modifications for the analysis of SA, JA, and ABA: ~40 mg of the tissue/sample ground in liquid nitrogen was extracted with 1.00 ml of 40 mM sodium phosphate buffer (pH 7.0). A 10 ng aliquot of d4-SA (C/D/N Isotopes Inc., Quebec, Canada, part #D-1156), 50 ng of d5-JA (C/D/N Isotopes Inc., part #D-6936), and 50 ng of d6-ABA (OlChemIm, Ltd., Olomouc, Czech Rebuplic, part #0342722) were added into each sample as internal standards. Samples were buffer-extracted at 4 °C on a lab rotator for 20 min, centrifuged at 12000 *g* for 15 min, and supernatants were collected and transferred to fresh 1.7 ml centrifuge tubes. The pH of supernatants was then adjusted using HCl, and samples were further purified via solid-phase extraction. Eluted samples were dried under nitrogen gas, re-dissolved in 100 µl of methanol, and filtered through 0.2 µm PTFE filters (Fisher Scientific, Pittsburgh, PA, USA part #03-391-4E). 

For LC-MS/MS analysis, 1 µl of each re-dissolved sample was injected into an Agilent 1260 infinity LC system. Compounds were separated using an Agilent Poroshell 120EC-C18 (3.5 × 50 mm, 2.7 µm) column and an acidified water:methanol buffer system (Buffer A: 0.1% acetate, 5% methanol in water; Buffer B: 0.1% acetate in methanol). Gradient conditions were as follows: hold at 2% B for 1.5 min, 2 min at 2–60% B, 4.5 min at 60–98% B, hold at 98% B for 3.5 min, and then back to 2% B in 1 min. Eluted samples were further separated and quantified through the coupled Agilent 6460 triple quadrupole dual mass spectrometer equipped with an electrospray ionization (ESI) source. Compounds were quantified in negative ion mode. ESI source parameters were set as follows: gas temperature at 250 °C, gas flow rate at 10 L min^–1^, nebulizer at 60 psi, sheath gas temperature at 400 °C, sheath gas flow at 12 L min^–1^, capillary at 4500 V, nozzle voltage at 500 V. Retention and mass transitions for SA, JA, and ABA were verified using authentic standards. Specific mass transitions (precursor ion→product ion pairs, *m/z*) monitored for each phytohormone were: ABA, 263→153, 263→203; JA, 209→59; and SA, 137→93, 137→65.

### qRT-PCR analysis

Three leaf samples of 6- to 7-week-old plants (~100 mg) per genotype were harvested before and at 5 dpi with *Gc* UCSC1 infection. Total RNA was isolated for each sample using TRIzol^®^ Reagent and reverse transcribed using SuperScript™ III Reverse Transcriptase (Invitrogen, Thermo Fisher Scientific Inc.). For each experiment, qRT-PCR was performed with three biological replicates per treatment and three technical replicates per sample using an Applied Biosystems 7300 Real-Time PCR System with SYBR™ Green PCR Master Mix (Thermo Fisher Scientific Inc.). The transcript levels of the target genes were normalized to that of *UBC9* (Ubiquitin conjugating enzyme 9, *AT4G27960*). Data were analyzed using the Applied Biosystems 7300 Real-Time PCR System Software and comparative ∆∆Ct method (Livak and Schmittgen, 2001). Primers are listed in Supplementary Table S2.

### JA sensitivity assay

The assay for Arabidopsis root response to MeJA was adapted from a previous report (Xiao *et al.*, 2004). Images of the seedlings were taken at day 10, and root length was measured using ImageJ (Schneider *et al.*, 2012).

## Results

### *PLDα1* and *PLDδ* play opposing roles in post-penetration resistance

**Fig. 1.**
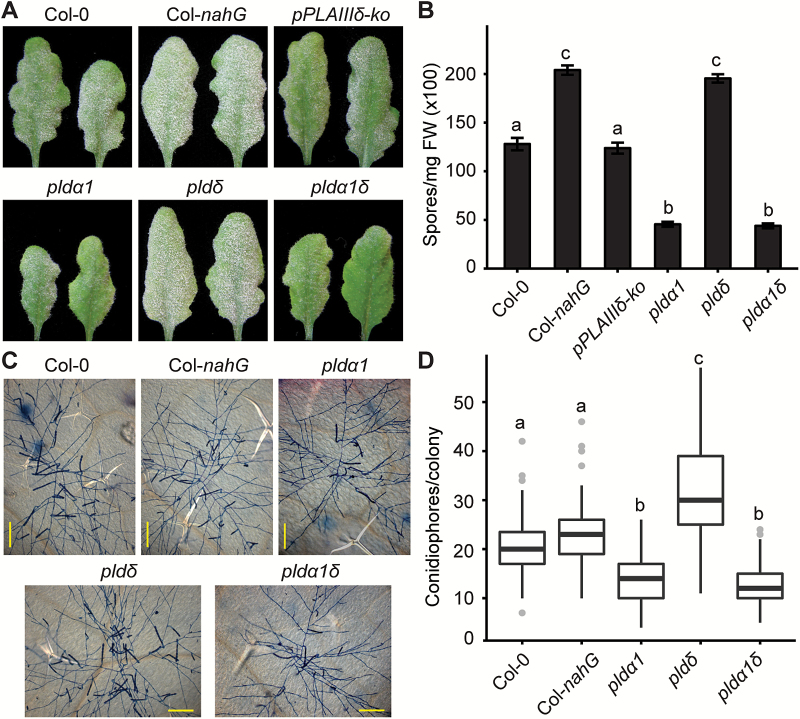
Arabidopsis *PLDα1* negatively modulates while *PLDδ* positively modulates post-penetration resistance against well-adapted powdery mildew *Gc* UCSC1. (A) Representative images of Arabidopsis leaves of the indicated genotypes infected with *Gc* UCSC1 at 12 dpi. Note, *pldα1* and *pldα1δ* were less susceptible while *pldδ* was more susceptible than Col-0. (B) Quantification of spore production in the indicated genotypes at 10 dpi normalized to leaf FW. Data represent the mean ±SEM of three samples (*n*=3, four leaves each) from one experiment, which was repeated three times with similar results. (C) Representative microscopic images of single colonies of *Gc* UCSC1 on leaves of the indicated genotypes at 4 dpi. Fungal structures were stained by trypan blue. Scale bars=200 μm. (D) Total number of conidiophores per colony on leaves of the indicated genotypes at 4 dpi. The boxplot shows combined data from three independent experiments (at least 20 colonies were counted for each genotype per experiment). The bold line within the box represents the median. The bottom and top edge of the box represent the first and third quartile, respectively. Ends of whiskers represent the minimum and maximum of data points. Gray dots represent outliers. Different lower case letters indicate statistically different groups (*P*<0.01) as determined by multiple comparisons using one-way ANOVA, followed by Tukey’s HSD test.

We tested a panel of T-DNA insertion lines (Supplementary Table S1) including six *PLD* knockout mutants (*pldα1*, *pldδ*, *pldβ1*, *pldα1δ*, *pldα1δα3*, and *pldα1δε*) with *Gc* UCSC1, a well-adapted powdery mildew isolate. Interestingly, we found that the *pldδ* mutant with compromised penetration resistance (Pinosa *et al.*, 2013) showed clear enhanced disease susceptibility (‘eds’) while *pldα1* defective in ABA signaling (Zhang *et al.*, 2004) and *pldα1*-containing mutants (*pldα1δ*, *pldα1δα3*, and *pldα1δε*) exhibited enhanced disease resistance (‘edr’) to *Gc* UCSC1 (Fig. 1A, B; Supplementary Fig. S1). The ‘edr’ phenotype of *pldα1δ* led us to speculate that *PLDα1* may act genetically downstream of *PLDδ* to modulate plant immunity negatively. Visual scoring of fungal mass on the leaf surface at 12 dpi and quantification of fungal spore production showed that the level of the ‘eds’ of *pldδ* was almost comparable with that of Col-*nahG*, a Col-0 transgenic line defective in SA signaling due to conversion of SA to catechol by the bacterial SA hydrolase encoded by *nahG* as a transgene (Fig. 1A, B). All other mutants tested exhibited levels of disease susceptibility similar to those of the Col-0 wild type (Fig. 1A, B; Supplementary Fig. S1). Consistent with the results at 12 dpi, *pldδ* supported significantly more conidiophores per colony while *pldα1* and *pldα1δ* had fewer conidiophores per colony than Col-0 during early infection stage at 4 dpi when the fungus begins asexual reproduction (Fig. 1C, D). Interestingly, Col-*nahG* supported a similar amount of conidiophores to Col-0 at 4 dpi (Fig. 1D), suggesting that PLDδ-mediated defense against *Gc* UCSC1 probably occurs earlier than SA-mediated defense. This raises an intriguing question as to whether PLDδ (and PLDα1) functions in a signaling pathway distinct from the SA-dependent pathway.

To test whether the ‘edr’ phenotype of *pldα1* and the ‘eds’ phenotype of *pldδ* are indeed due to the loss of *PLDα1* and *PLDδ*, respectively, multiple *pldα1* and *pldδ* lines expressing the respective wild-type genes were generated and tested with *Gc* UCSC1. These lines displayed similar disease phenotypes to Col-0 (Supplementary Fig. S2), indicating genetic complementation of these two genetic mutations by their respective wild-type genes. Thus, our genetic data established a positive role for *PLDδ* and a negative role for *PLDα1* in basal, stage II post-penetration resistance against well-adapted powdery mildew in Arabidopsis.

**Fig. 2.**
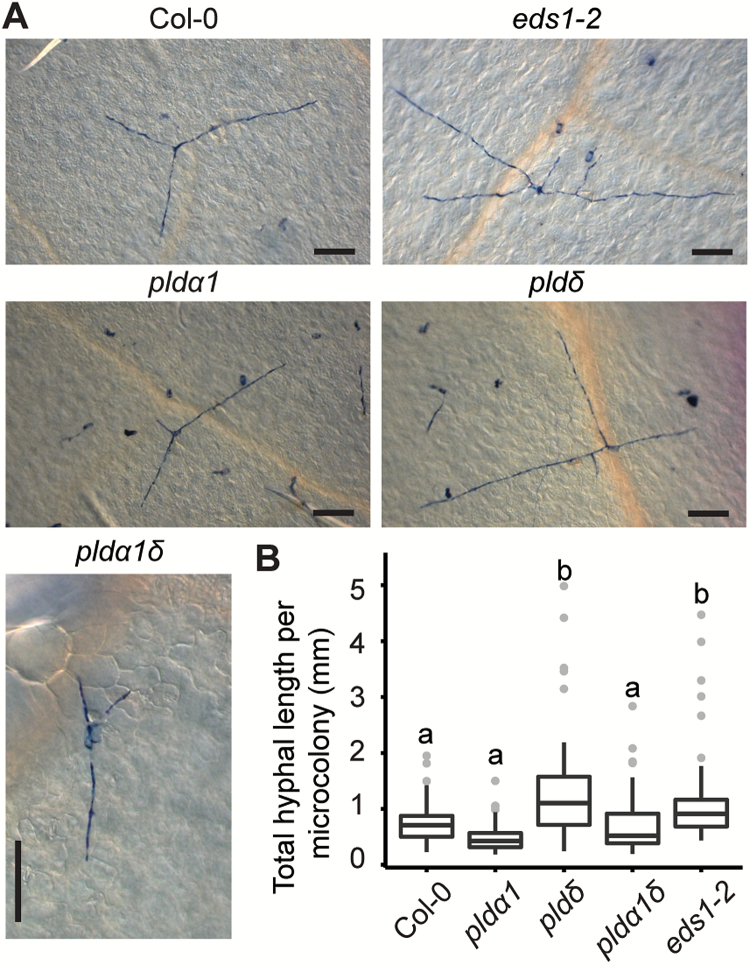
*PLDδ* in Arabidopsis contributes to post-penetration resistance against a non-adapted powdery mildew *Gc* UMSG1. (A) Representative microscopic images of typical *Gc* UMSG1 fungal microcolonies grown on leaves of the indicated genotypes at 5 dpi. Scale bars=100 μm. (B) Total hyphal length per microcolony of the indicated genotypes at 5 dpi. The boxplot shows combined data from three independent experiments (*n* >60). Different lower case letters indicate statistically different groups as determined by multiple comparisons using one-way ANOVA, followed by Tukey’s HSD test (*P*<0.01).

To test if the *PLD* genes are also involved in stage I post-penetration resistance, we inoculated the *pld* mutants with *Gc* UMSG1. *Gc* UMSG1 is a powdery mildew fungus infectious on sow thistle. It has largely overcome penetration resistance of 25 Arabidopsis accessions examined and is capable of forming initial haustoria but arrested before sporulation by stage I post-penetration resistance in Arabidopsis (Wen *et al.*, 2011). We assessed the growth of *Gc* UMSG1 on the *pld* mutants by measuring the total hyphal length of each microcolony at 5 dpi. Not surprisingly, *pldδ* supported significantly more hyphal growth than Col-0 (Fig. 2B), which is similar to *eds1-2* (in Col-0; Bartsch *et al.*, 2006), known to support better growth of *Gc* UMSG1 (Wen *et al.*, 2011). However, while limited sporulation of *Gc* UMSG1 can occasionally be seen on *eds1-2*, indicating breakdown of non-host resistance, it has never been observed on *pldδ*, suggesting that *PLDδ* acts differently from *EDS1* and is not as critical as *EDS1* in stage I post-penetration resistance defined by this pathosystem. However, hyphal growth in *pldα1* and *pldα1δ* showed no significant difference from that in Col-0 (Fig. 2).

The subcellular defense responses such as powdery mildew-induced H_2_O_2_ production and callose deposition were investigated in the *pld* mutants. Because *Gc* UCSC1 can largely suppress the production of H_2_O_2_ in Col-0 (Xiao *et al.*, 2005), the non-adapted isolate *Gc* UMSG1 was used to challenge the plants, and *in situ* H_2_O_2_ production was visualized by DAB staining (Thordal-Christensen *et al.*, 1997). We divided the haustorium–epidermal cell interaction in terms of H_2_O_2_ production into three types: (i) H_2_O_2_ is undetectable; (ii) H_2_O_2_ accumulates in the haustorial complex; and (iii) H_2_O_2_ is found in both the haustorial complex and the whole cell (Supplementary Fig. S3A). Of >750 interaction sites evaluated in Col-0, 39.5, 25.7, and 34.7% were (i), (ii), and (iii), respectively, and the *pld* mutants showed a similar frequency distribution for the three interaction types (Supplementary Fig. S3B). This suggests that H_2_O_2_ production induced by haustorium invasion is not affected due to loss of *PLDα1* or *PLDδ*, or both. Next, we examined callose deposition at the fungal penetration sites (i.e. papillae) or around the haustorium (i.e. haustorial encasement) by aniline blue staining after *Gc* UCSC1 inoculation. Again, callose deposition was grossly unaffected in the *pld* mutants compared with that in Col-0 based on visual scoring (Supplementary Fig. S3C). These suggest that the ‘eds’ phenotype of *pldδ* and the ‘edr’ phenotype of *pldα1* are not apparently associated with these two typical subcellular defense responses.

### Loss of *PLDα1* or *PLDδ* affects basal resistance against an oomycete but not ETI

**Fig. 3.**
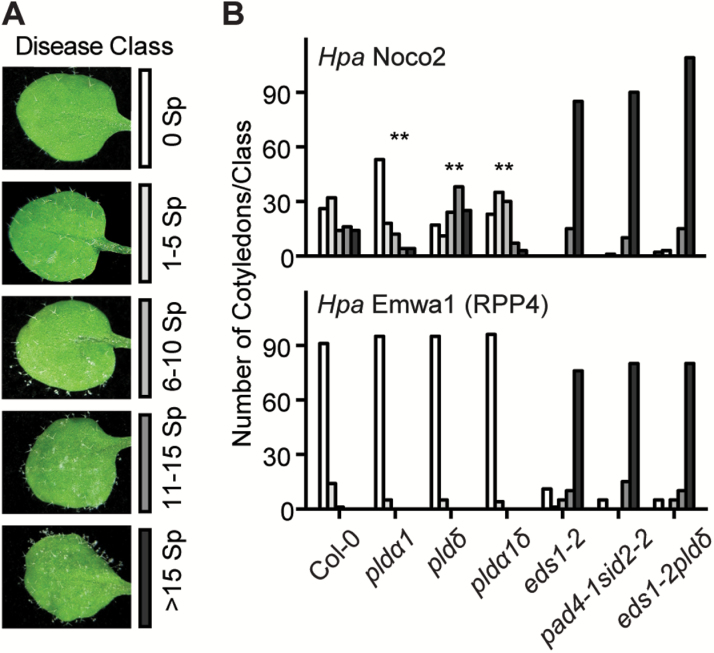
Loss of *PLDα1* and/or *PLDδ* affects basal resistance against oomycetes, but not ETI mediated by RPP4. (A) Representative cotyledons showing disease phenotypes of the indicated disease classes at 7 dpi. Ten-day-old seedlings were inoculated with virulent *Hyaloperonospora arabidopsidis* (*Hpa*) isolate Noco2 or avirulent isolate Emwa1. Sporangiophores (Sp) per cotyledon were assessed at 7 dpi, and categorized into five classes as indicated by the corresponding figure keys. (B) Quantification of the number of cotyledons (*n* >100 for each of the indicated genotypes) per class of the indicated genotypes infected with Hpa isolate Noco2 (upper panel) or avirulent isolate Emwa1 (lower panel) based on categorization of leaf infection defined in (A). χ^2^ test was used to test statistical significance for disease degree between Col-0 and the indicated mutant lines at 7 dpi (**P<0.01).

*Hyaloperonospora arabidopsidis* (*Hpa*) is a fungus-like oomycete pathogen of Arabidopsis. To test if post-penetration resistance to *Hpa* is also altered in the *pld* mutants, we inoculated 10-day-old seedlings of Col-0, *pldα1*, *pldδ*, *pldα1δ*, and two known ‘eds’ mutant lines, *eds1-2* and *pad4-1sid2-2*, with *Hpa* isolate Noco2 (virulent on Col-0). While *pldα1* and *pldα1δ* were significantly less susceptible, *pldδ* was significantly more susceptible (albeit not as susceptible as *eds1-2* and *pad4-1sid2-2*) to this pathogen than Col-0 (*P*<0.01) (Fig. 3B, upper panel). These further support the distinct roles of *PLDα1* and *PLDδ* in post-penetration resistance against haustorium-forming pathogens.

To test if loss of *PLDα1* or *PLDδ* impacts ETI, we tested the mutants with an avirulent oomycete strain *Hpa* Emwa1 (recognized by *RPP4*, a TIR-NB-LRR; van Der Biezen *et al.*, 2002), and *Pseudomonas syringae* pv. *maculicola* (*Pma*) ES4326 strains expressing either AvrRpm1 (recognized by RPM1, a CC-NB-LRR; Grant *et al.*, 1995) or AvrRps4 (recognized by RPS4/RRS1, a pair of TIR-NB-LRR immune receptors; Narusaka *et al.*, 2009), since no *NB-LRR*-mediated resistance against powdery mildew has been defined in Arabidopsis. While *eds1-2* and *pad4-1sid2-2* were compromised in resistance against *Hpa* Emwa1, the *pld* mutants displayed similar levels of resistance to that seen in Col-0 (Fig. 3), indicating that loss of *PLDα1* and/or *PLDδ* does not seem to affect *RPP4*-dependent ETI. Similarly, no significant difference was detected between *pldα1*, *pldδ*, *pldα1δ*, and Col-0 (Supplementary Fig. S4C, D) in defense against *Pma*, further supporting that *PLDα1* or *PLDδ* individually or together do not play a significant role in ETI. In addition, the *pld* mutants remained resistant like Col-0 to *Pma ∆hrcC*, which is unable to inject type III effectors to suppress PTI, implying that the PTI against bacterial pathogens is not affected by the loss of *PLDα1* and/or *PLDδ* (Supplementary Fig. S4B). This could be due to functional redundancy among the PLD enzymes in defense against bacterial pathogens as suggested in an earlier study since there are 12 PLD isoforms in Arabidopsis (Johansson *et al.*, 2014).

### PLDδ is dispensable for RPW8-mediated resistance

*RPW8.1* and *RPW8.2* (referred to as *RPW8* in later text unless otherwise indicated) confer post-penetration, haustorium-targeted resistance to powdery mildew (Xiao *et al.*, 2001; Wang *et al.*, 2009). To examine whether PLDα1 and/or PLDδ contribute to RPW8-mediated resistance, we first stably expressed the *RPW8.2-RFP* (red fluorescent protein) transgene from the native *RPW8.2* promoter in *pldα1* and *pldδ*. Confocal microscopy showed that the localization of RPW8.2–RFP to the EHM was unchanged in *pldα1* or *pldδ* (as represented by RPW8.2–RFP’s localization in *pldδ*; Supplementary Fig. S5A), indicating that neither PLDα1 nor PLDδ is required for precise EHM targeting of RPW8.2 (Wang *et al.*, 2009). Next, we individually introduced these two mutations into S5 (a Col-*gl* line expressing *RPW8*; Xiao *et al.*, 2005). Both S5/*pldα1* and S5/*pldδ* displayed the same levels of resistance to *Gc* UCSC1 (Supplementary Fig. S5C, D) and H_2_O_2_ production as S5 in haustorium-invaded cells (as represented by H_2_O_2_ production in S5/*pldδ*,; Supplementary Fig. S5B). Given that RPW8’s defense function requires SA signaling (Xiao *et al.*, 2005), these results support that the PLDα1/PLDδ pair most probably function via an SA-independent signaling pathway.

### PLDα1 and PLDδ have distinct subcellular localizations

**Fig. 4.**
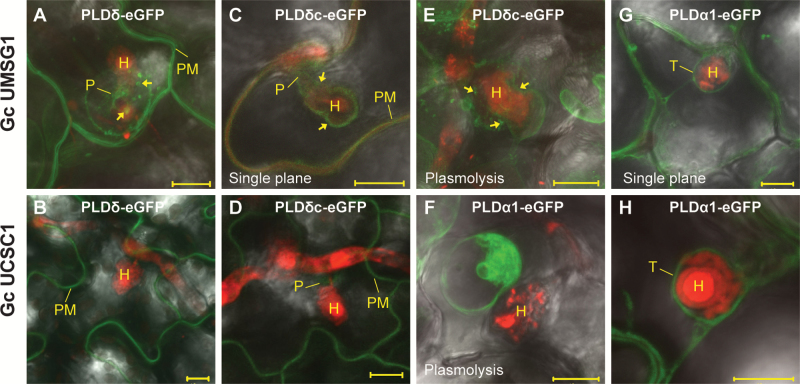
Differential subcellular localization of PLDα1 and PLDδ in powdery mildew-infected epidermal cells. Stable transgenic lines were inoculated with *Gc* UMSG1 or *Gc* UCSC1. At 2 dpi, sections of infected leaves were immersed in propidium iodide (PI, 0.5% aqueous solution) for 40–60 min for staining haustoria (H, red) and mycelia (red) before confocal imaging. All representative images shown are merged (GFP, PI, and bright field) *Z*-stack projections of 15–20 optical sections unless otherwise indicated. (A, B) Localization of PLDδ–eGFP (from the *PLDδ* genomic sequence translationally fused with *eGFP*) in a *Gc* UMSG1-invaded cell (A) or a *Gc* UCSC1-invaded cell (B). Arrows: concentric ring and dots. (C–E) Localization of PLDδc-eGFP (from the *PLDδ* full-length coding sequence translationally fused with *eGFP*; Pinosa *et al.*, 2013) in a *Gc* UMSG1-invaded cell before (C; arrows, peri-haustorial membrane) or after plasmolysis (E; 0.5 M NaCl for 20 min; arrows indicate dots and membrane retained around the haustorium), or a *Gc* UCSC1-invaded cell (D). (F–H) Localization of PLDα1–eGFP in a *Gc* UMSG1-invaded cell (G), or a *Gc* UCSC1-invaded cell before (H) or after plasmolysis (F). Scale bars=10 μm. PM, plasma membrane; P, penetration site; T, tonoplast.

Since there is active membrane trafficking and biogenesis (of the EHM) in haustorium-invaded cells (Berkey *et al.*, 2017), we wondered whether the contrasting defense responses of *pldα1* and *pldδ* to adapted powdery mildew are due to possible differential subcellular enzymatic activities of PLDα1 and PLDδ in haustorium-invaded cells. To test this, we fused *eGFP* to the C-termini of the genomic DNA of the two *PLD* genes and expressed the fusion constructs from *35S* plus the native promoter (for *PLDα1-eGFP*) or *35S* (for *PLDδ-eGFP*) in *pldα1* or *pldδ*, respectively, since the GFP signal from the native promoter-driven *PLDδ* cDNA (*PLDδc*) in fusion with *eGFP* was reported to be too weak for imaging (Pinosa *et al.*, 2013). *PLDδ-eGFP* could fully, while *PLDα1-eGFP* could partially, rescue the respective mutant phenotypes (Supplementary Fig. S6), indicating that these fusion proteins are (partially) functional. We then used leaves of the respective transgenic lines infected with *Gc* UMSG1 or *Gc* UCSC1 at 2 dpi for subcellular localization analysis using confocal microscopy. When examining leaves infected with *Gc* UMSG1, we detected PLDδ–eGFP in the PM of all epidermal cells and in two or more concentric rings around the penetration site forming the ‘bull’s eye’ domain (Assaad *et al.*, 2004; Koh *et al.*, 2005) often with small dots or bulbs within or nearby (Fig. 4A). However, it was rarely seen in the *Gc* UCSC1 penetration site (Fig. 4B), implying that the adapted pathogen suppresses the recruitment of PLDδ–eGFP to the probably perturbed PM around the papilla. PLDδc–eGFP was reported to exhibit focal accumulation around the *Bgh* penetration site in Arabidopsis epidermal cells (Pinosa *et al.*, 2013). We thus examined the subcellular localization of the PLDδc–eGFP expressed from *35S* in our pathosystems. In the case of *Gc* UMSG1, PLDδc–eGFP was often more preferentially detected in the ‘bull’s eye’ domain (Fig. 4A) or in an EHM-like membrane surrounding the constrained haustorium than PLDδ–eGFP (Fig. 4C). After plasmolysis (0.5 M NaCl for 20 min), GFP signal was retained around the haustorium in small dots or bulbs (Fig. 4E), similar to those in the papilla at the penetration site (Fig. 4A), indicating that PLDδc–eGFP is not at the EHM because the EHM largely remains intact within 30 min of such plasmolysis treatment (Berkey *et al.*, 2017). In the case of *Gc* UCSC1, the PLDδc–eGFP signal was much weaker at the penetration site (Fig. 4D), suggesting that recruitment of PLDδc–eGFP to the penetration site is also similarly suppressed by the adapted powdery mildew pathogen. The slight discrepancy in localization between PLDδ–eGFP and PLDδc–eGFP may be attributable to alternative splicing of *PLDδ* (Wang and Wang, 2001) which is pertinent to the *PLDδ-eGFP* construct but irrelevant to the *PLDδc-eGFP* construct for which a full-length *PLDδ* cDNA was used (Pinosa *et al.*, 2013).

A strong fluorescence signal of PLDα1–eGFP was found in a peri-haustorium membrane similar to the EHM (Fig. 4G, H), which could be completely separated from the haustorium after plasmolysis (Fig. 4F). This indicates that PLDα1–eGFP is not localized to the EHM but rather it may be in the tonoplast that tightly wraps around the haustorium.

These results in general agree with the subcellular localizations of PLDα1 and PLDδ inferred by protein localization and fractionation analyses in earlier studies (Wang, 2000; Wang and Wang, 2001; Pinosa *et al.*, 2013). The distinct localization patterns of these two PLDs may in part contribute to their opposing roles in post-penetration resistance against powdery mildew pathogens.

### PLDδ contributes to resistance independent of EDS1/PAD4, SA, and JA signaling pathways

Our earlier results (Fig. 1C, D; Supplemenary Figs S3, S5) suggest that PLDδ and perhaps PLDα1 may function through an SA-independent pathway. To define this pathway further, we made double and triple mutants by crossing *pldα1* or *pldδ* to well-characterized SA-dependent (*sid2-2*) (Wildermuth *et al.* 2001, Dewdney *et al.* 2000) or both SA-dependent and -independent signaling (*eds1-2* and *pad4-1*) mutants (Bartsch *et al.*, 2006; Venugopal *et al.*, 2009).

We first examined if *pldδ*-mediated ‘eds’ is additive to the ‘eds’ phenotypes of *eds1-2* or *pad4-1* in response to the well-adapted *Gc* UCSC1 isolate and found that *eds1-2pldδ* and *pad4-1pldδ* were not statistically more susceptible than the single mutants (Supplementary Fig. S7A, B). We then made *pad4-1sid2-2pldδ*, *eds1-2pad4-1pldδ*, and *eds1-2pad4-1sid2-2* triple mutants, and compared the disease phenotypes between these and the two double mutants. No significant differences were detected between the mutants except *pad4-1sid2-2pldδ* versus *pad4-1sid2-2* (Supplementary Fig. S7A, B), suggesting that either PLDδ somehow acts in the SA pathway or the *pldδ*-mediated ‘eds’ phenotype may be masked in the various double or triple mutants because *Gc* UCSC1 is too aggressive on these mutants to allow reliable detection of any phenotypic differences.

**Fig. 5.**
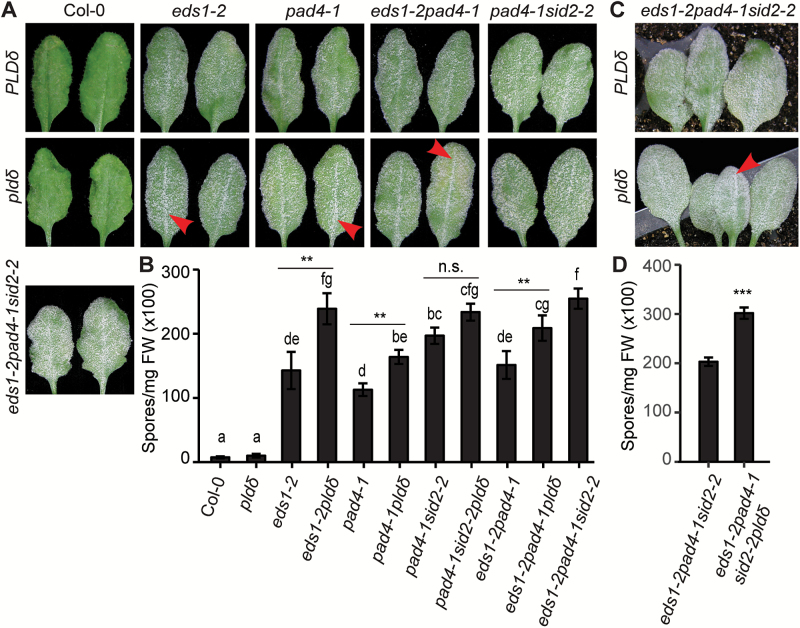
*PLDδ* in Arabidopsis contributes to post-penetration resistance via an SA- and EDS1/PAD4-independent pathway.(A, C) Representative leaves of the indicated genotypes (defined by name IDs from both *x*- and *y*-axes) infected with *Gc* UMSG3 at 11 dpi. Note that fungal mass is more noticeable on leaves, especially the mid-vein area (arrowheads), from *eds1-2pldδ*, *pad4-1pldδ*, *eds1-2pad4-1pldδ*, and *eds1-2pad4-1sid2-2pldδ* than the corresponding leaves from *eds1-2*, *pad4-1*, *eds1-2pad4-1*, and *eds1-2pad4-1sid2-2* (upper panel). (B, D) Quantification of spore production in the indicated genotypes in (A, C), respectively, at 11 dpi normalized to leaf FW. Data represent the mean ±SEM of four samples (*n*=4, 4–5 leaves each) from one experiment, which was repeated three times with similar results. Different lower case letters indicate statistically different groups as determined by multiple comparisons using one-way ANOVA, followed by Tukey’s HSD test (B, **P<0.01), or by Student’s *t*-test (D, ***P<0.001). n.s., not significant.

To test the latter possibility, we used *Gc* UMSG3, a powdery mildew isolate from tobacco which can only weakly sporulate on Col-0, to resolve subtle infection phenotypic differences between different genotypes. Sporulation of *Gc* UMSG3 was found to be very weak on both Col-0 and *pldδ*; however, a whitish fungal mass was more easily discernible on *pldδ* at 11 dpi (Fig. 5A, B). Interestingly, *eds1-2*, *pad4-1*, *eds1-2pad4-1*, and *pad4-1sid2-2* all supported profuse sporulation (Fig. 5A), suggesting that EDS1 and/or PAD4 make a major contribution to stage II post-penetration resistance to *Gc* UMSG3 probably via both SA-dependent and SA-independent mechanisms.

Notably, *eds1-2pldδ* and *pad4-1pldδ* supported significantly more fungal growth (white powder around the mid-vein in particular) than *eds1-2* and *pad4-1* visually (Fig. 5A) and quantitatively (Fig. 5B), indicating that PLDδ contributes to resistance against *Gc* UMSG3 through a mechanism(s) that is at least partially EDS1 or PAD4 independent. Interestingly, *pad4-1sid2-2* was as susceptible as *pad4-1pldδ* (Fig. 5B), which seemingly implies that PLDδ and SID2 may act in the same signaling pathway. Yet, *pad4-1sid2-2pldδ* was significantly more susceptible than *pad4-1pldδ* to *Gc* UMSG1 (Fig. 5A, B) and *pad4-1sid2-2* to *Gc* UCSC1 (Supplementary Fig. S7A, B). Similarly, *eds1-2pad4-1pldδ* exhibited an even higher level of susceptibility than *eds1-2pad4-1* and *pad4-1pldδ* (Fig. 5A, B). Finally, *eds1-2pad4-1sid2-2pldδ* exhibited significantly higher susceptibility to *Gc* UMSG3 than *eds1-2pad4-1sid2-2* (Fig. 5C, D). These observations together support that PLDδ acts through a yet to be characterized pathway to limit fungal infection at the post-penetration stage. It is worth pointing out that *eds1-2pldδ* showed a similar level of susceptibility to *eds1-2pad4-1pldδ* (Fig. 5A, B), implying that EDS1 and PAD4 are both required for resistance against *Gc* UMSG3. Supporting this inference, *eds1-2pad4*-1 was not statistically more susceptible than *eds1-2* or *pad4-1* (Fig. 5A, B).

To assess if PLDδ functions through the JA pathway, the *Gc* UCSC1 infection phenotype of *pldδ* was compared with that of *dde2-2,* which is impaired in JA biosynthesis (von Malek *et al.*, 2002). The susceptibility of *dde2-2* was similar to that of Col-0 (Supplementary Fig. S7C, D), consistent with our earlier finding that the JA signaling receptor mutant *coi1* did not show ‘eds’ to *Gc* UCSC1 (Xiao *et al.*, 2005), suggesting that the JA pathway has little or very limited contribution to defense against *Gc* UCSC1. Taken together, PLDδ is unlikely to act through the JA pathway.

Next, we investigated if the ‘edr’ phenotype of the *pldα1* mutant is affected by the *sid2-2*, *eds1-2*, or *pad4-1* mutations by first crossing *pldα1* to the three single and *pad4-1sid2-2* double mutants and then testing their infection phenotypes. Intriguingly, *eds1-2pldα1*, *pad4-1pldα1*, *sid2-2pldα1*, and *pad4-1sid2-2pldα1* all displayed similar ‘eds’ to *Gc* UCSC1 to the respective single or double mutants with wild-type *PLDα1* (Supplementary Fig. S8). This suggests that *pldα1*-mediated ‘edr’ is completely neutralized/suppressed when the SA- and/or EDS1/PAD4-mediated signaling is defective, genetically placing PLDα1 upstream of EDS1, PAD4, and SID2, which is in sharp contrast to the epistatic effect of *pldα1*-mediated ‘edr’ over *pldδ*-caused ‘eds’. A mechanistic model is proposed to explain the distinct yet related roles of PLDδ and PLDα1 (see the Discussion; Supplementary Fig. S9).

### Loss of *PLDα1* and/or *PLDδ* has no significant impact on SA, JA, and ABA levels and signaling

**Fig. 6.**
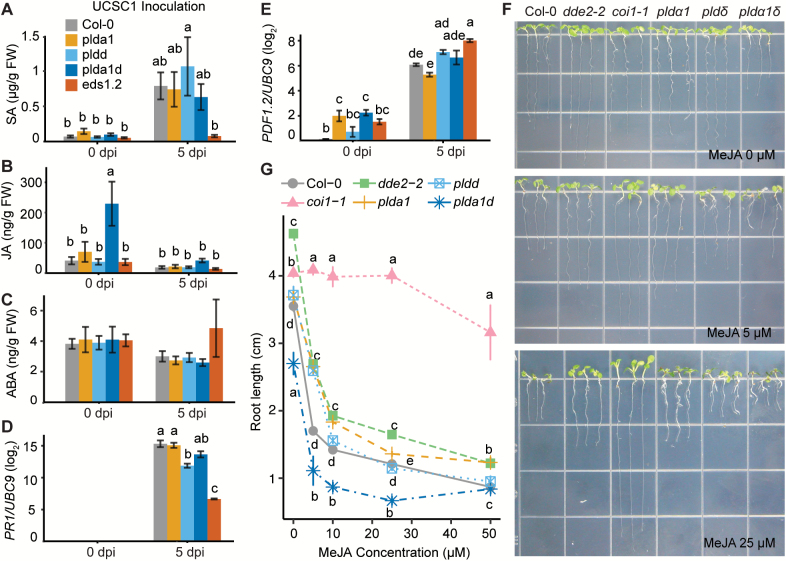
Impact of the *pldα1* and *pldδ* single and double mutations on the levels and signaling of SA and JA before and after powdery mildew infection. (A–C) Levels of the plant hormones SA (A), JA (B), and ABA (C) were measured by LC-MS/MS in leaves of 6-week-old plants of the indicated genotypes prior to (0 dpi) and post- (5 dpi) *Gc* UCSC1 inoculation. Notably, before inoculation, the JA level of *pldα1δ* was higher than that of the two single mutants and was reduced by ~4-fold at 5 dpi. Bars represent the mean ±SEM of three independent experiments combined (*n*=3 for each experiment). (D, E) Log2-fold changes of *PR1* (D) or *PDF1.2* (E) relative to *UBC9* encoding ubiquitin conjugating enzyme 9. Bars represent the mean ±SEM of three biological replicates. (F) Representative pictures showing 10-day-old seedlings of the indicated genotypes grown on MS-agar medium without or with 5 μM and 25 μM MeJA. (G) Dose–response curve of root growth of the indicated genotypes upon MeJA treatment. Root lengths of 10-day-old seedlings growing on MS-agar medium supplemented with exogenous MeJA at 0, 5, 10, 25, or 50 μM were measured and are presented as the mean ±SEM at each MeJA dosage. The line graph shows combined data from two independent experiments (*n* >15 for each experiment). Different lower case letters indicate statistically different groups (*P*<0.05) as determined by multiple comparisons using one-way ANOVA, followed by Tukey’s HSD test.

To investigate if PLDα1- and/or PLDδ-mediated defense mechanisms are connected with defense-related phytohormones, we first measured levels of endogenous SA, ABA, and JA in *pldα1*, *pldδ*, and *pldα1δ* along with Col-0 and *eds1-2* prior to and at 5 dpi with *Gc* UCSC1 using LC-MS/MS. Compared with naïve plants, SA levels increased by 5- to 16-fold in mildew-infected Col-0 and *pld* mutants, but remained low in *eds1-2* (Fig. 6A), indicating that pathogen-induced SA biosynthesis is intact in the *pld* mutants. To see if SA signaling is affected in the *pld* mutants, the expression of the marker gene *PR1* (Wiermer *et al.*, 2005) was measured and found to be induced to a level similar to that in Col-0, suggesting that SA signaling was not affected by any of the *pld* mutations (Fig. 6D). These results support the inference from our genetic data that PLDα1 and PLDδ oppositely modulate post-penetration resistance via an SA-independent pathway. No significant changes in ABA levels were observed in Col-0 and the *pld* mutants before and after powdery mildew infection (Fig. 6C).

Surprisingly, the JA level in uninfected *pldα1δ* was higher (3- to 6-fold) than that in all other genotypes (Fig. 6B), and the expression of its marker gene *PDF1.2* was significantly higher in unchallenged *pldα1* and *pldα1δ* compared with that in Col-0 (Fig. 6E), suggesting that PLDα1 and PLDδ may act together to repress JA production/signaling in the absence of pathogens. At 5 dpi with *Gc* UCSC1, JA in *pldα1δ* was reduced to a level that is only slightly higher (~2-fold) than that in other plants (Fig. 6B), which is probably caused by an antagonistic effect from enhanced SA biosynthesis and signaling in the mildew-infected plants. However, despite a slight decrease in JA levels in all the genotypes at 5 dpi, expression levels of *PDF1.2* showed a similar increase (2.5- to 12-fold ) in all the plants, with no significant difference between the *pld* mutants and Col-0 (Fig. 6E). Together these results indicate that (i) although well-adapted powdery mildew infection does not induce JA biosynthesis, it can still induce JA signaling; and (ii) the altered defense phenotypes in *pldα1* and *pldα1δ* do not correlate with the changes in JA levels and/or JA signaling.

It is known that high JA levels inhibit root growth (Staswick *et al.*, 1992). To test the results concerning the endogenous JA levels further, we examined root growth of *pldα1δ* along with Col-0, *pldα1*, *pldδ*, and two JA mutants, *dde2-2* (defective in JA synthesis; von Malek *et al.*, 2002) and *coi1-1* (insensitive to JA; Xie *et al.*, 1998) in Murashige and Skoog (MS)-agar medium without or with supplement of exogenous methyl jasmonate (MeJA). Consistent with the results from the JA level measurements, only roots of *pldα1δ* grown in MeJA-free MS-agar medium were significantly shorter (~76.9% of Col-0) (Fig. 6F, G). Roots of all genotypes, except those of *coi1-1*, showed similar rates of growth inhibition in MS-agar medium supplemented with different concentrations of MeJA (5, 10, 25, and 50 µM) (Fig. 6G). This indicates that JA signaling in the *pld* mutants is not affected. Taken together, our results further demonstrate that PLDα1 and PLDδ oppositely modulate defense in an SA-independent manner but may act together to curb JA accumulation in naïve plants.

## Discussion

In this study, we collected genetic evidence to demonstrate that Arabidopsis PLDα1 and PLDδ oppositely modulate basal, post-penetration resistance against powdery mildew, and oomycete pathogens via an EDS1/PAD4-, SA-, and JA-independent pathway.

### PLDδ and PLDα1 modulate post-penetration resistance against powdery mildew

Pinosa *et al*. previously reported that the loss-of-function *pldδ* mutant is compromised in penetration resistance against the non-adapted barley mildew *Bgh* (Pinosa *et al.*, 2013). Here, we show that the same *pldδ* mutant exhibited ‘eds’ to a well-adapted powdery mildew isolate *Gc* UCSC1 (Fig. 1) and supported more hyphal growth of the non-adapted powdery mildew isolate *Gc* UMSG1 that has overcome penetration resistance (Wen *et al.*, 2011) (Fig. 2). This implies that the PLDδ-based defense mechanism operates throughout the entire infection cycle of powdery mildew and apparently has not been (fully) suppressed by even aggressive powdery mildew pathogens such as *Gc* UCSC1. To determine if PLDδ-mediated defense is effective against other pathogens, we tested *pldδ* with the fungus-like oomycete *Hpa* Noco2 that also employs a haustorium-based nutrient acquisition strategy. Notably, *pldδ* was significantly more susceptible than Col-0 but not as susceptible as *eds1-2* or *pad4-1sid2-2* to *Hpa* (Fig. 3B). Given that powdery mildew fungi only invade host epidermal cells while oomycete pathogens invade both epidermal and mesophyll cells (Takemoto *et al.*, 2003), it is possible that PLDδ-mediated defense is more effective in epidermal cells compared with mesophyll cells. It is also possible that oomycete pathogens may be able to suppress PLDδ-mediated defense more effectively than powdery mildew. In addition, PLDδ-mediated defense may be attenuated under higher humidity (>90%) conditions necessary for infection of *Hpa* Noco2. High humidity-caused suppression of resistance has been reported for several different defense mechanisms (Xiao *et al.*, 2003; Zhou *et al.*, 2004; Wang *et al.*, 2007). Similar to what was reported earlier (Johansson *et al.*, 2014), we did not observe any difference in growth of virulent bacteria between Col-0 and *pldδ*, suggesting that PLDδ is specifically involved in defense against cell wall-breaching pathogens. Notably, among all reported genes involved in penetration and post-penetration resistance, PLDδ is unique in that it contributes to both penetration and post-penetration resistance against powdery mildew fungi. In contrast to *pldδ*, both the *pldα1* single and the *pldα1δ* double mutant exhibited ‘edr’ to virulent powdery mildew and oomycete pathogens (Figs 1, 3). This suggests that genetically *PLDα1* and *PLDδ* function oppositely in the same pathway with *PLDα1* acting downstream of *PLDδ*. We reported earlier that loss of *PLDβ1* resulted in ‘edr’ to virulent bacterial pathogens and ‘eds’ to a necrotrophic fungal pathogen *Botrytis cinerea* (Zhao *et al.*, 2013), suggesting a positive role for PLDβ1 in the JA pathway and a negative role in the SA pathway. We found in this study that *pldβ1* showed slight ‘edr’ to *Gc* UCSC1 based on our visual scoring of the infection phenotypes (Supplementary Fig. S1A), supporting a role for PLDβ1 in modulating SA–JA signaling. Whether PLDα1 and PLDβ1 share similar regulatory mechanisms and/or have overlapping function remains to be determined.

### PLDα1 and PLDδ may modulate defense via a potentially novel pathway

Three lines of genetic evidence collectively support our conclusion that PLDδ functions through an SA-independent pathway. First, RPW8-mediated resistance, which is known to engage SA signaling, is intact in *pldδ* (Fig. S5C); secondly, adding the *pldδ* mutation to the SA signaling mutants *eds1-2* and *pad4-1*, or the SA biosynthesis mutant *sid2-2*, resulted in increased ‘eds’ to the poorly adapted isolate *Gc* UMSG3 (Fig. 5); lastly, *pldδ* showed similar elevation of SA levels and induction of *PR1* expressions to Col-0 upon powdery mildew infection (Fig. 6A, D).

Because EDS1 and PAD4 are believed to function upstream of SA and modulate defense via both SA-dependent and SA-independent pathways (Bartsch *et al.*, 2006; Venugopal *et al.*, 2009), the increased ‘eds’ of *eds1-2pldδ*, *pad4-1pldδ*, *eds1-2pad4-1pldδ*, and *eds1-2pad4-1sid2-2pldδ* to *Gc* UMSG3 (Fig. 5) also provide clear genetic evidence to support a role for PLDδ in defense through an EDS1- and/or PAD4-independent pathway. However, based on our genetic analyses alone, we could not exclude the possibility that PLDδ also contributes to EDS1/PAD4-dependent resistance. It is possible that the defense pathways mediated by EDS1, PAD4, and PLDδ may be interconnected or partially overlapping, since the phenotypic differences among the single and double mutants concerning these three genes were largely diminished when they were tested with the aggressive isolate *Gc* UCSC1 (Supplementary Fig. S7).

We also evaluated whether PLDα1 and PLDδ function via the JA pathway. Our results from genetic analysis (Supplementary Fig. S7C, D; Xiao *et al.*, 2005), measurements of JA levels (Fig. 6B), and *PDF1.2* expression (Fig. 6E) showed that the altered defense phenotypes of the *pld* mutants could be uncoupled from the changes in the JA levels and signaling, thus excluding the possibility that PLDα1 and PLDδ modulate defense through the JA pathway.

Taken together, our results indicate that PLDα1 and PLDδ play opposing roles in modulating resistance against powdery mildew via a pathway that is independent of the EDS1/PAD4, SA, and JA pathways. Notably, *mlo*-based durable and broad-spectrum resistance against powdery mildew has recently been shown to be independent of all the known defense pathways (Kuhn *et al.*, 2017). Therefore, it will be interesting for future studies to determine if PLDα1 and PLDδ have a mechanistic connection with MLO or other known defense pathways such as the ET and mitogen-activated protein (MAP) kinase signaling pathways (Tsuda *et al.*, 2013; Kim *et al.*, 2014; Hillmer *et al.*, 2017; Kuhn *et al.*, 2017).

### PLDα1 may repress PLDδ-mediated defense signaling

We previously reported that PLDα1 promotes H_2_O_2_ production whereas PLDδ facilitates downstream H_2_O_2_ signaling in guard cells to regulate stomatal closure positively during drought stress (Zhang *et al.*, 2009; Guo *et al.*, 2012). However, our genetic data from this study position *PLDα1* as a negative regulator downstream of *PLDδ*-mediated defense. Consistent with this, powdery mildew haustorium-induced H_2_O_2_ production was not affected in any of the *pld* mutants (Supplementary Fig. S3A, B). Given that drought response relies on the movement of guard cells, whereas plant defense against powdery mildew pathogens mostly occurs in the leaf pavement cells, it is possible that the proteins interacting with these two PLDs and/or their substrates during drought stress and pathogen infection are different. Hence, it is conceivable that PLDα1 and PLDδ probably participate in distinct signaling networks in these two different types of cells in response to abiotic and biotic stresses.

It is unclear to us how PLDδ positively modulates while PLDα1 negatively modulates post-penetration resistance against powdery mildew pathogens. One possible mechanism is that PLDα1 and PLDδ exert their opposing roles in defense by producing distinct pools of PA to modulate distinct cellular processes by targeting spatiotemporally restricted proteins at different subcellular localizations (Supplementary Fig. S9). Our confocal microscopy show that while PLDδ–eGFP is localized at the PM, around the penetration site and peri-haustorium, PLDα1–eGFP is most likely to be associated with the tonoplast and other intracellular membranes (Fig. 4), which are compatible with results previously reported (Wang, 2000; Wang and Wang, 2001; Pinosa *et al.*, 2013). Notably, the eGFP signal of PLDδ–eGFP was the strongest around the penetration site of non-host barley mildew (Pinosa *et al.*, 2013), weaker around the penetration site and/or the haustorial complex of the non-adapted *Gc* UMSG1, and almost undetectable in such subcellular compartments induced by the well-adapted *Gc* UCSC1 (Fig. 4A–D). This suggests that PLDδ is recruited to the PM around the penetration site to produce PA to (in)activate target proteins locally, and adapted powdery mildew pathogens may suppress this recruitment to interfere with PLDδ’s role in defense activation. As for PLDα1, its tonoplast localization may be related to vacuole-based removal of defense molecules to prevent inappropriate activation of defense in the absence of pathogens. However, its suppression is relieved by PLDδ-triggered signaling once pathogens invade. Future work will be directed to identifying relevant immunity proteins that are modulated by the two functionally distinct PLDs.

## Supplementary data

Supplementary data are available at *JXB* online.

Fig. S1. Disease reaction phenotypes of *pPLA*, *PLD*, *PLC*, *DGK*, and *PIP5K* T-DNA insertion mutants infected with *Gc* UCSC1.

Fig. S2. Genetic complementation of the *pldα1* and *pldδ* mutant genes by their respective wild-type genes.

Fig. S3. Loss of *PLDα1*, *PLDδ*, or both does not impact H_2_O_2_ production and callose deposition in the haustorium-invaded epidermal cells.

Fig. S4. Loss of *PLDα1* and/or *PLDδ* does not affect ETI against bacterial pathogens.

Fig. S5. *PLDα1* and *PLDδ* are not required for RPW8-mediated resistance to *Gc* UCSC1.

Fig. S6. The PLDδ–eGFP and PLDα1–eGFP fusion proteins are functional.

Fig. S7. *Gc* UCSC1 infection phenotypes of *pldδ*-containing double and triple mutants and relevant controls.

Fig. S8. The ‘edr’ phenotype of *pldα1* to *Gc* UCSC1 is suppressed by the *eds1-2*, *sid2*-2, and/or *pad4-1* mutations.

Fig. S9. A working model for the roles of PLDα1 and PLDδ in plant immunity.

Table S1. Arabidopsis T-DNA insertion mutants screened in this study.

Table S2. Primers used in this study.

Supplementary Figures and Tables

## Abbreviations:

Avr: avirulence factor
Bgh: *Blumeria graminis* f.sp. *hordei*
CC-NB-LRRs: coiled-coil–nucleotide-binding site–leucine-rich repeat
DAB: 3,3'-diaminobenzidine
DGK: diacylglycerol kinase
EDS1: ENHANCED DISEASE SUSCEPTIBILITY 1
EHM: extra-haustorial membrane
ET: ethylene
ETI: effector-triggered immunity
Gc: *Golovinomyces cichoracearum*
Hpa: *Hyaloperonospora arabidopsidis*
HR: hypersensitive response
JA: jasmonic acid
NB-LRR: nucleotide-binding site–leucine-rich-repeat
NDR1: NON-RACE-SPECIFIC DISEASE RESISTANCE 1
PA: phosphatidic acid
PAD4: PHYTOALEXIN-DEFICIENT 4
PAMP: pathogen-associated molecular pattern
PEN1: PENETRATION1
PIP5K: phosphatidylinositol 4-phosphate 5-kinase
PLC: phospholipase C
PLD: phospholipase D
PM: plasma membrane
Pma: *Pseudomonas syringae* pv. *maculicola*
pPLA: patatin-related phospholipase
PR: pathogenesis-related
PRR: pattern recognition receptor
PTI: PAMP-triggered immunity
SA: salicylic acid
TIR-NB-LRRs: Toll-interleukin 1 receptor–NB–LRRs
UBC9: ubiquitin conjugating enzyme 9.
