# Supplementary material for: Arabidopsis phospholipase Dα1 and Dδ oppositely modulate EDS1- and SA-independent basal resistance against adapted powdery mildew
Source: J Exp Bot. 2018 Apr 18;69(15):3675–88. doi: 10.1093/jxb/ery146 (PMC6022666; doi:10.1093/jxb/ery146)
Supplement: Supplementary Figures and Tables [file ery146_suppl_supplementary_figures_tables.pdf]

Supplementary Figure S1

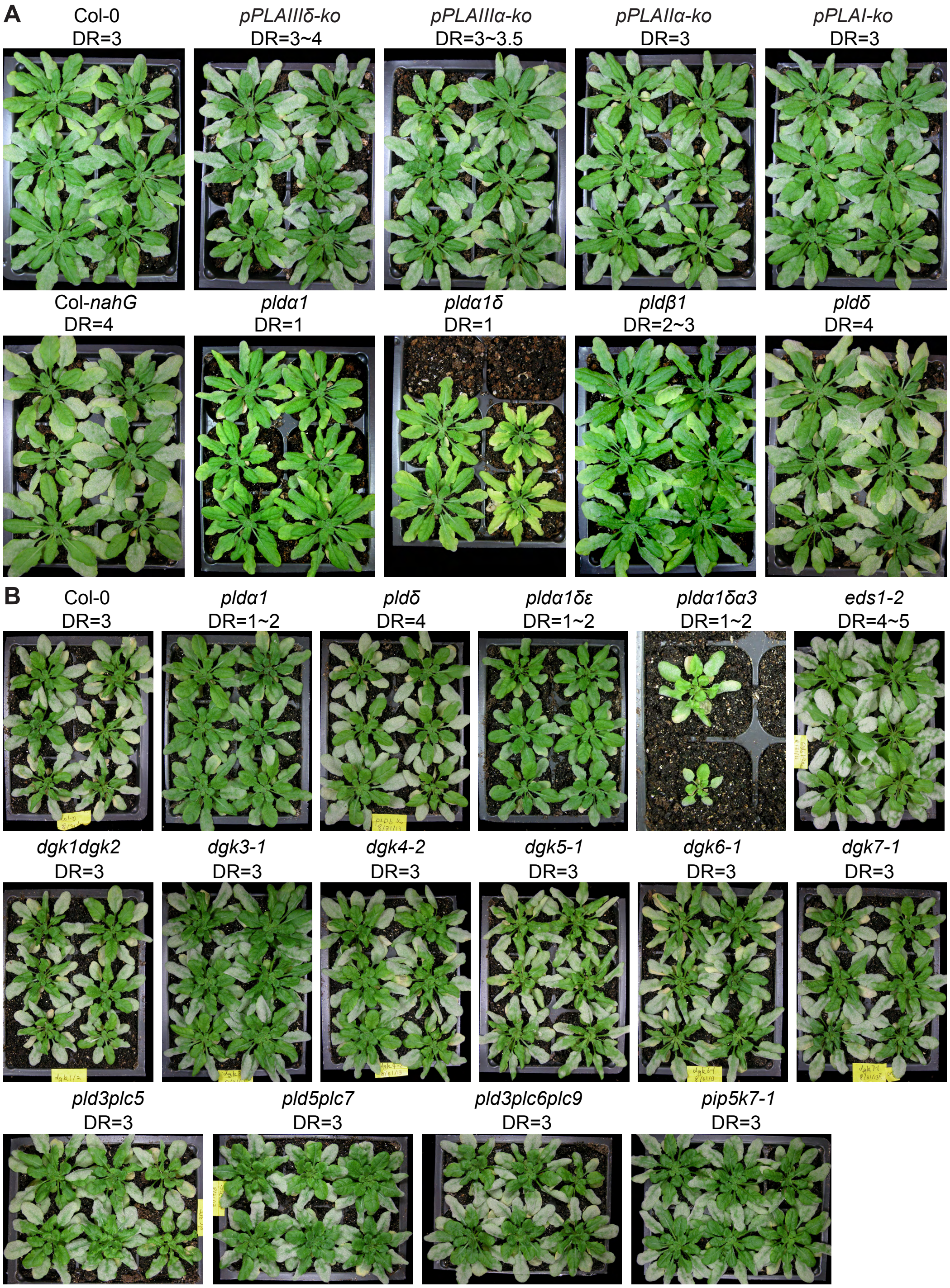

Supplementary Figure S1. Disease reaction phenotypes of *pPLA*, *PLD*, *PLC*, *DGK* and *PIP5K* T-DNA insertion mutants infected with *Gc* UCSC1.
(A, B) Representative plants of indicated genotypes infected with *Gc* UCSC1 at 8 dpi **(A)** or 11 dpi **(B)**. Note that *pldβ1* showed slight "edr" to *Gc* UCSC1, and *pldα1δα3* and *pldα1δε* triple mutants displayed similar level of "edr" as *pldα1*. The disease reaction (DR) scores (0, resistant; 1 to 2, intermediate; 2 to 3 or 3 or 3 to 4, susceptible; 4 to 5, "eds" ; Xiao et al., 2005) are shown above the photos.

Supplementary Figure S2

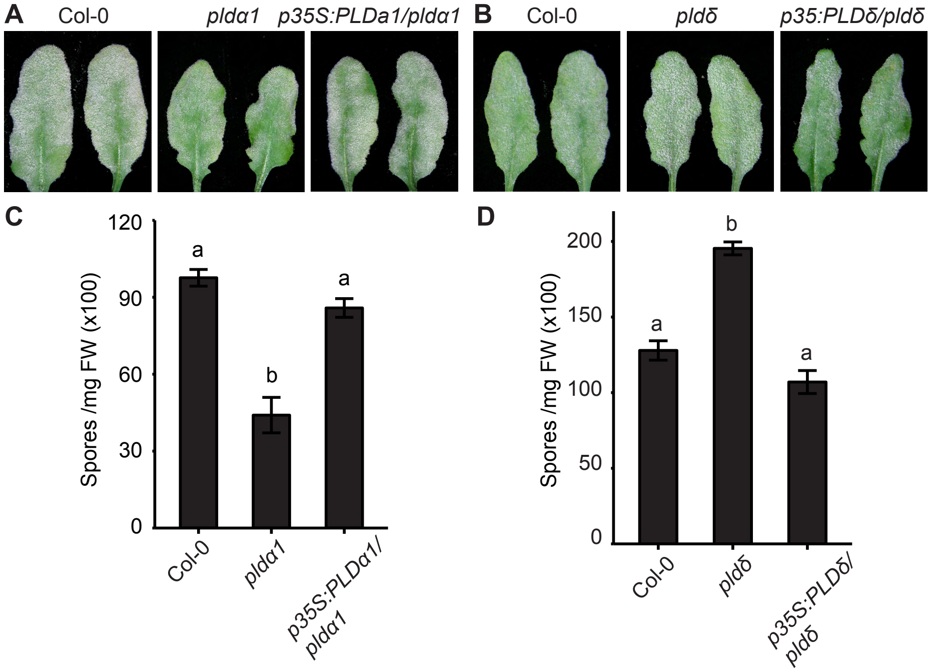

Supplementary Figure S2. Genetic complementation of the *pldα1* and *pldδ* mutant genes by their respective wild-type genes.
(A, B) Representative images of leaves of indicated genotypes infected with *Gc* UCSC1 at 13 and 10 dpi, respectively.
(C, D) Quantification of spore production in leaves of indicated genotypes from **(A, B)** normalized to leaf fresh weight (FW). Data represent mean ± SEM of four samples in **(A)** and three samples in **(B)** (4 leaves each sample), from one experiment, which was repeated twice with similar results. Different lowercase letters indicate statistically different groups as determined by multiple comparisons using one-way ANOVA, followed by Tukey-HSD ($P < 0.01$).

Supplementary Figure S3

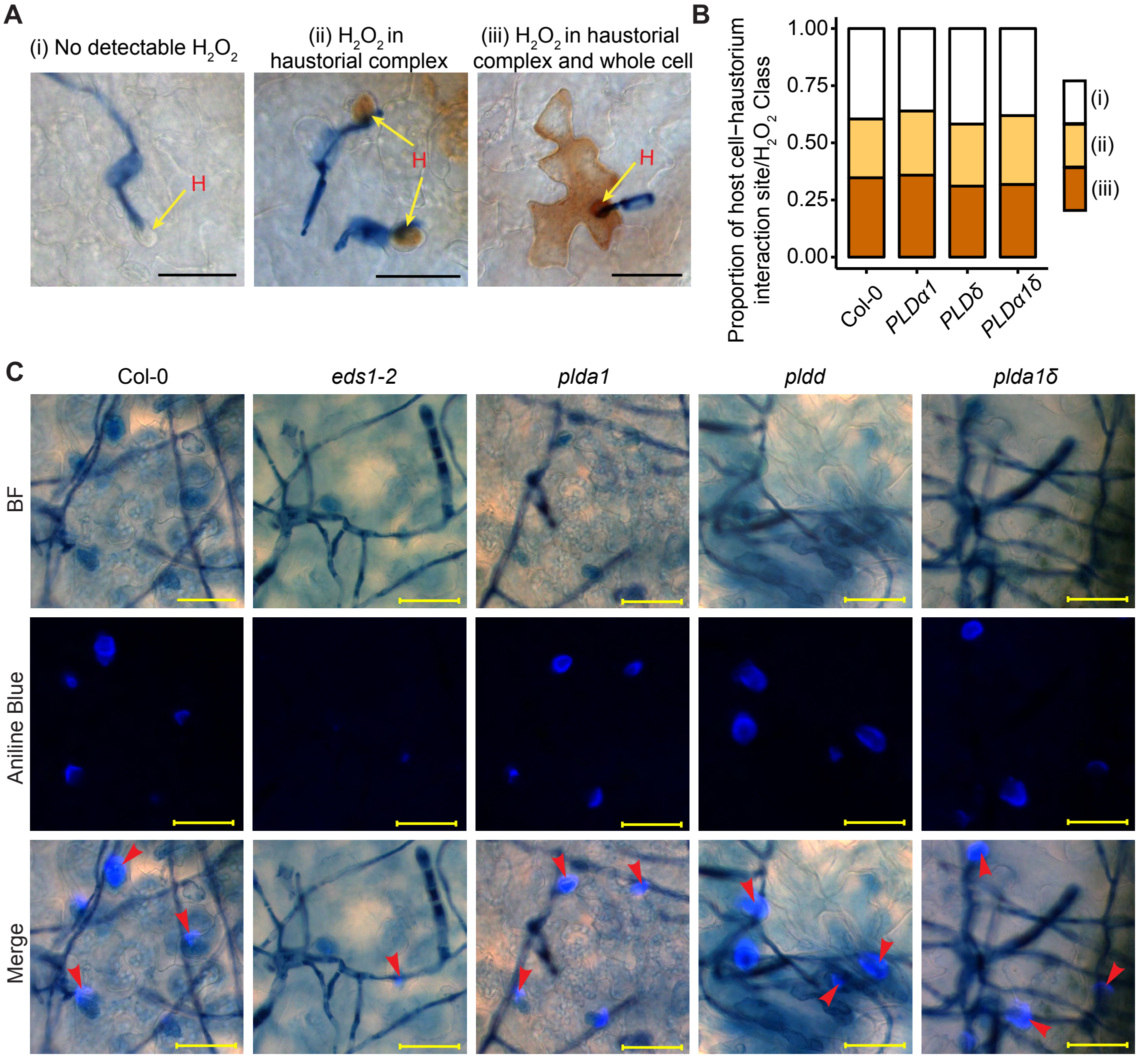

Supplementary Figure S3. Loss of *PLDα1* or *PLDδ* or both do not impact H_2O_2 production and callose deposition in the haustorium-invaded epidermal cells.

(A) Representative images of three types of H_2O_2 production in haustorium-epidermal cell interaction site: (i) H_2O_2 is not detectable; (ii) H_2O_2 accumulates in the haustorial complex; and (iii) H_2O_2 is found in both haustorial complex and the whole cell. Leaf samples were inoculated with *Gc* UMSG1 and stained by 3,3'-diaminobenzidine (DAB) at 3dpi.

(B) Frequencies of the three types of H_2O_2 production shown in **(A)** in each of the indicated genotypes. Total of between 750 to 1300 interaction sites combined from three independent experiments were evaluated for each genotype.

(C) Representative images showing callose formation in the indicated genotypes. Leaf samples were inoculated with *Gc* UCSC1 and stained blue by aniline blue at 3dpi. Arrowheads indicate three types of callose deposition: encasement of the haustorium, half encasement of the haustorium, and callose is restricted to the penetration site. Bars, 50 µM. BF, bright field.

Supplementary Figure S4

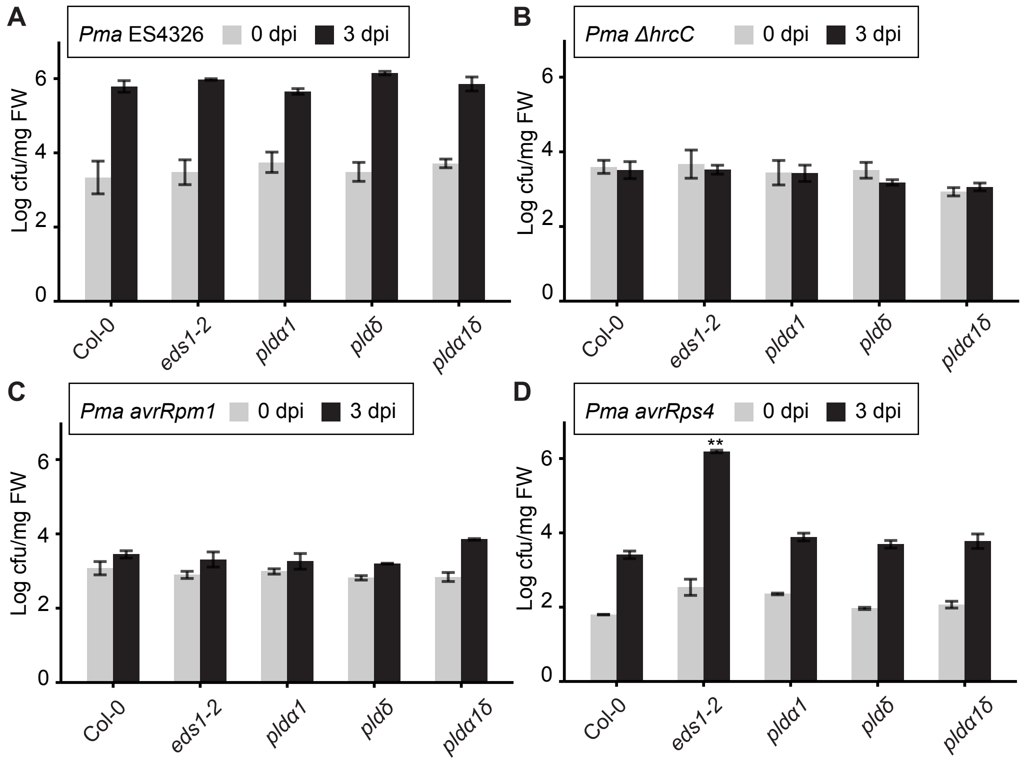

Supplementary Figure S4. Loss of *PLDα1* and/or *PLDδ* does not affect ETI against bacterial pathogens.

Fifteen-day-old seedlings were dip-inoculated with *Pma* ES4326 **(A)**, *Pma ΔhrcC* **(B)**, *Pma avrRpm1* **(C)** and *Pma avrRps4* **(D)**. Seedling samples were collected at 0 dpi (1 hour post inoculation) and 3 dpi and bacterial growth was quantified. Data represent mean ± SEM (n = 4). One-way ANOVA followed by Tukey-HSD was conducted to evaluate whether there was any significant difference in bacterial growth between Col-0 and the indicated genotypes (**$p < 0.01$, $p>0.05$ for the remaining). FW, fresh weight.

Supplementary Figure S5

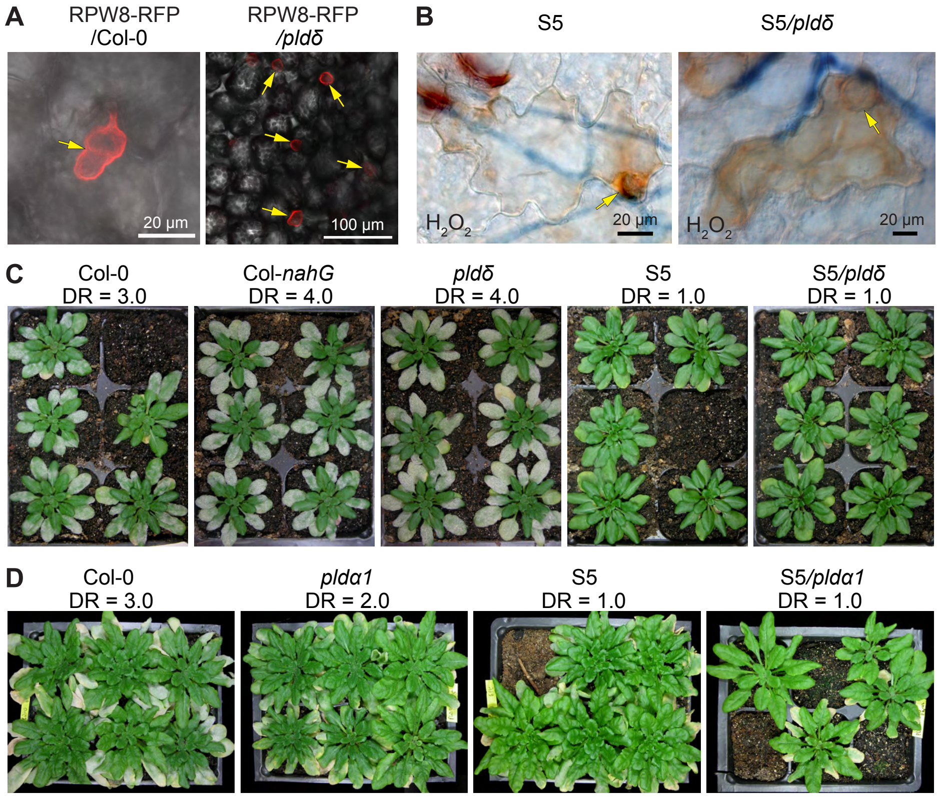

Supplementary Figure S5. *PLDα1* and *PLDδ* are not required for RPW8-mediated resistance to *Gc* UCSC1.
(A) Subcellular localization of RPW8-RFP in Col-0 and *pldδ* in *Gc* UCSC1 haustorium-invaded cells. The confocal images shown are Z-stack projections of 15 optical sections taken at 2 dpi. Note that RPW8-RFP localization in *pldδ* mutant was not affected.
(B) RPW8-triggered H_2O_2 accumulation in haustorium-invaded epidermal cells of S5 (Col-0 expressing RPW8) and S5/*pldδ* was visualized by 3,3'-diaminobenzidine (DAB) staining at 3 dpi with *Gc* UCSC1. Haustoria are indicated by arrows. Bars, 20µm.
(C, D) Representative plants of indicated genotypes infected with *Gc* UCSC1 at 13 dpi **(C)** and 14 dpi **(D)**. The disease reaction (DR) scores (0, resistant; 1 to 2, intermediate; 2 to 3 or 3 or 3 to 4, susceptible; 4 to 5, "eds" ; Xiao et al., 2005) are shown above the images.

Supplementary Figure S6

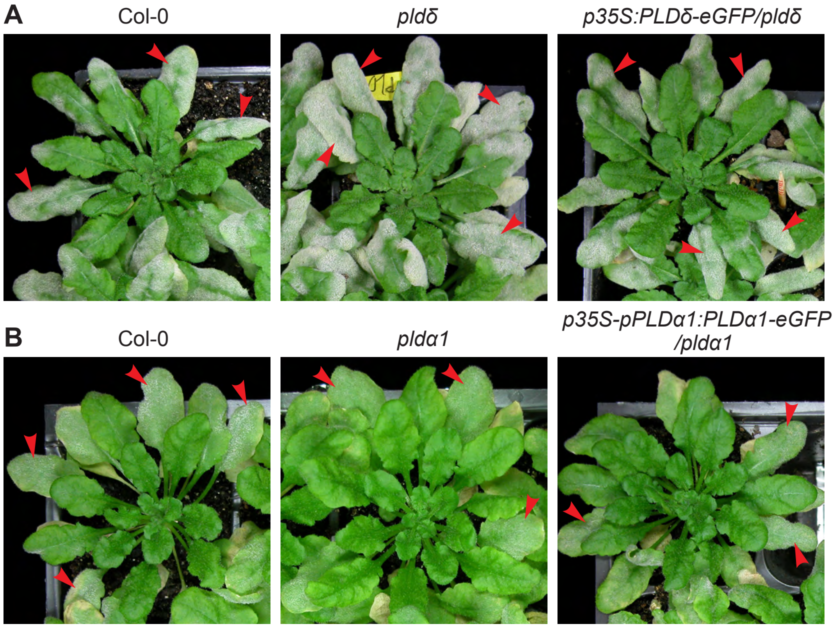

Supplementary Figure S6. The PLDδ-eGFP and PLDα1-eGFP fusion proteins are functional.
Representative plants of the indicated genotypes infected with *Gc* UCSC1 at 12dpi. While the transgene *35S:PLDδ-eGFP* could fully rescue the “eds” phenotype of *pldδ* **(A)**, *p35S-pPLDα1:PLDα1-eGFP* could partially restore the “edr” phenotype of *pldα1* **(B)**. Leaves marked with red arrowheads display typical disease phenotypes of indicated genotypes.

Supplementary Figure S7

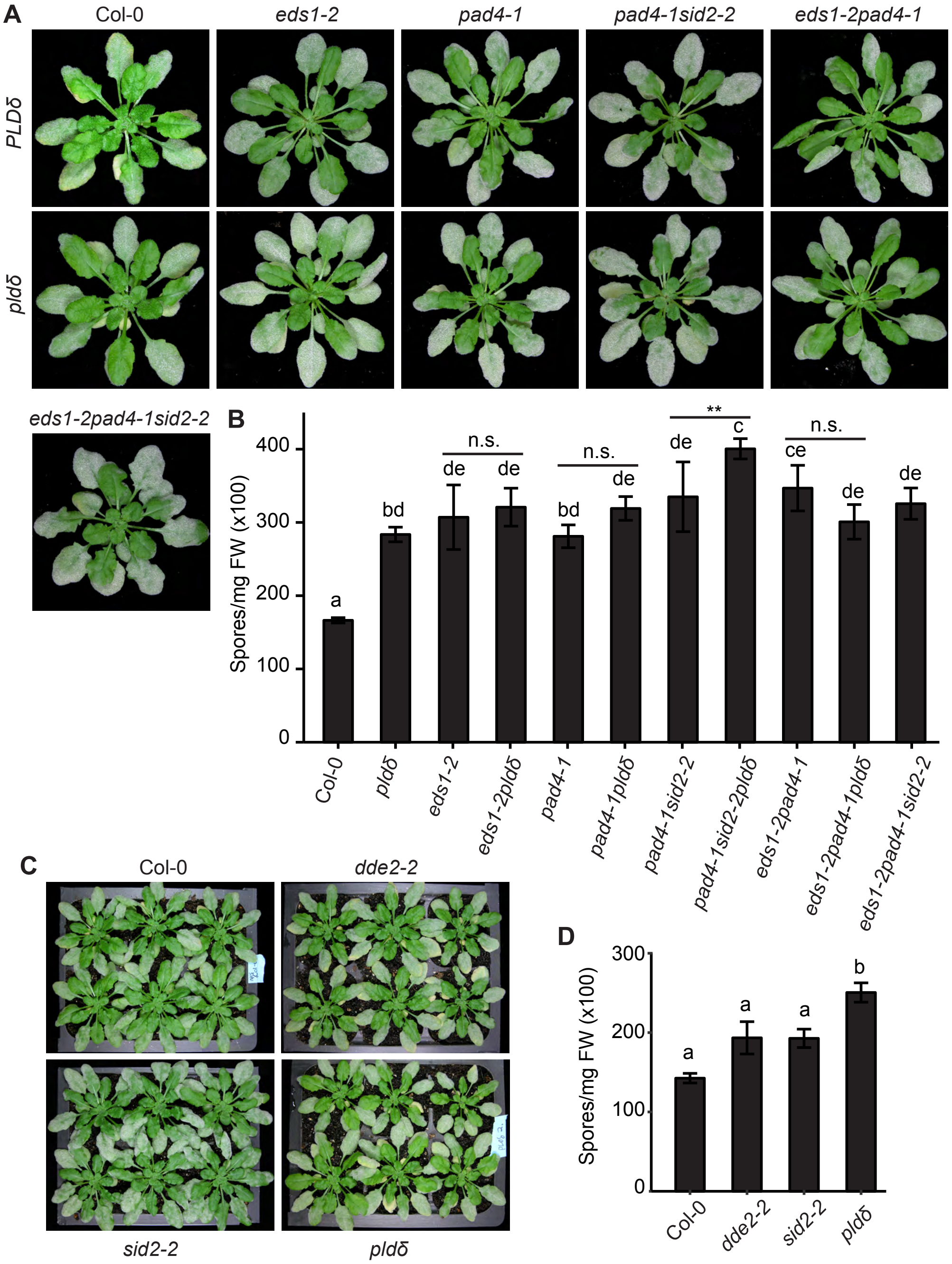

Supplementary Figure S7. *Gc* UCSC1 infection phenotypes of *pldδ*-containing double and triple mutants and relevant controls.
(A) Representative leaves of indicated genotypes (defined by name IDs from both X and Y axises) infected with *Gc* UCSC1 at 10 dpi.
(B) Quantification of spore production in indicated genotypes at 10 dpi normalized to leaf fresh weight (FW).
(C) Plants of indicated genotypes infected with *Gc* UCSC1 at 10dpi.
(D) Quantification of spore production of plants in **(C)**. Bars represent mean ± SEM of four samples (n=4, 4 leaves each) from one experiment, which was repeated three times with similar results. Different lowercase letters indicate statistically different groups as determined by multiple comparisons using one-way ANOVA, followed by Tukey-HSD (**P < 0.01). Note that no significant (n.s.) difference (P>0.05) was found in three of the four indicated pair of genotypes in **(B)**.

Supplementary Figure S8

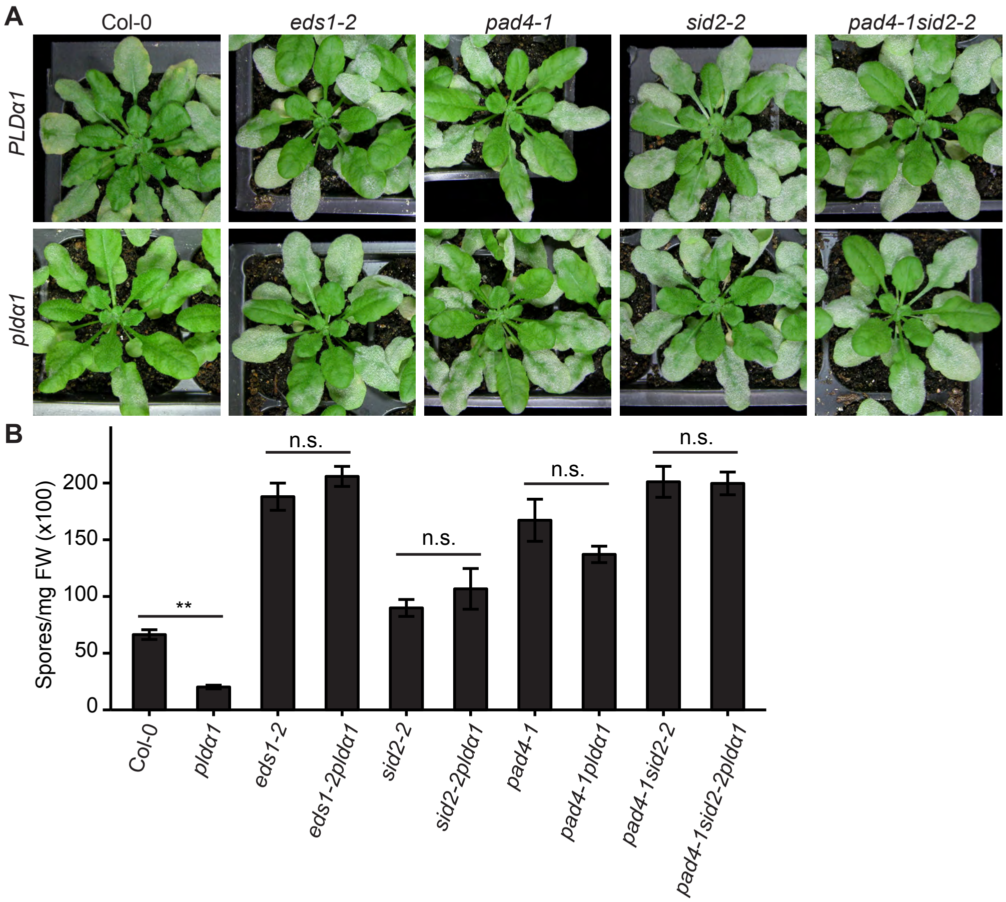

Supplementary Figure S8. The "edr" phenotype of *pldα1* to *Gc* UCSC1 is suppressed by the *eds1-2*, *sid2-2* and/or *pad4-1* mutations.
(A) Representative leaves of indicated genotypes (defined by name IDs from both X and Y axises) infected with *Gc* UCSC1 at 10 dpi.
(B) Quantification of spore production in indicated genotypes at 10 dpi normalized to leaf fresh weight (FW). Data represent mean ± SEM of four samples (n=4, 4 leaves each) from one experiment, which was repeated three times with similar results. No significant (n.s.) difference (P>0.05, Student *t*-test) was detected in all the pairs indicated, except for the Col-0, *pldα1* pair (**p<0.01).

Supplementary Figure S9

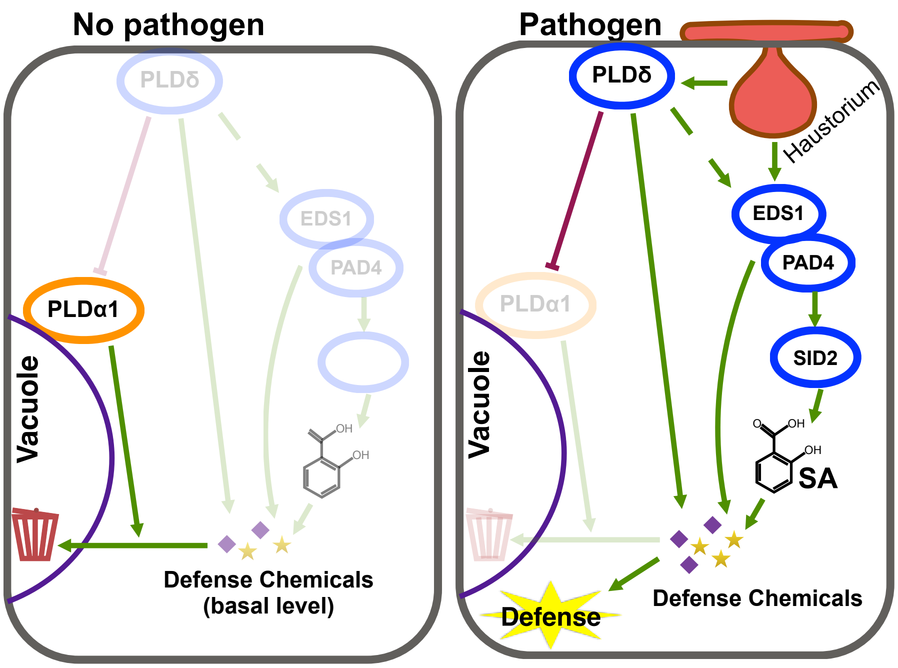

Supplementary Figure S9. A working model for the roles of PLDα1 and PLDδ in plant immunity.
In this model, PLDδ positively whereas PLDα1 negatively modulates plant basal resistance against powdery mildew with PLDα1 acting downstream of PLDδ. We hypothesize that upon perception of pathogen invasion, plasma membrane-associated PLDδ is activated and functions through a novel, SA-independent, signaling pathway(s), which is also distinct from, but possibly overlapping with, the EDS1/PAD4-dependent pathway(s) (indicated by a dashed line). By contrast, intracellular PLDα1 is involved in removal of defense chemicals produced from basal activities of PLDδ- and EDS1/PAD4-dependent pathways, thereby preventing inappropriate activation of defenses in the absence of pathogens. However, in the presence of powdery mildew or oomycete pathogens, PLDδ is activated, repressing PLDα1 activity, which leads to accumulation of defense chemicals, resulting in activation of defense responses. This model also implies that PA pools produced in different subcellular compartments have distinct roles in regulation of plant defense responses.

Table S1: Arabidopsis T-DNA insertion mutants screened in this study

Mutant name	Gene Locus	T-DNA line
*pld*α*1*[1]	At3g15730	SALK_053785
*pld*β*1*[2]	At2g42010	SALK_079133
*pld*δ[3]	At4g35790	SALK_023247
*pld*α*1*δ	At3g15730/At4g35790	see above
*pPLAIII*δ*-knockout (ko)*	At3g63200	SALK_029470
*pPLAIII*γ*-ko*	At4g29800	SALK_088404
*pPLAIII*β*-ko*[4]	At3g54950	SALK_057212
*pPLAIII*α*-ko*	At2g39220	SALK_040363
*pPLAII*δ*-ko*	At4g37060	SALK_090933
*pPLAII*β*-ko*	At4g37050	SALK_142351
*pPLAII*α	At2g26560	SALK_059119
pPLAI-ko	At1g61850	SALK_087152
pip5k7-1	At1g10900	SALK_151429c
plc3/5	At4g38530/At5g58690	SALK_037453/SALK_144469
plc5/7	At5g58690/At3g55940	SALK_144469/SALK_030333
plc3/6/9	At4g38530/At2g40116/At3g47220	SALK_037453/SALK_090508/SALK_025949
dgk1/2	At5g07920/At5g63770	SALK_053412/SAIL_ 718_G03
dgk3-1	At2g18730	SALK_028600
dgk4-2	At5g57690	SALK_069158
dgk5-1	At2g20900	SAIL_1212_E10
dgk6-1	At4g28130	SALK_016285
dgk7-1	At4g30340	SALK_51_E04
*pld*α*1*δα*3*	At3g15730/At4g35790/At5g25370	SALK_067533 / SALK_023247 / SALK_122059
*pld*α*1*δε	At3g15730/At4g35790/At1g55180	SALK_067533 / SALK_023247 /KONCZ68434

References

[1] Wenhua Zhang et al. "Phospholipase Dα1-derived phosphatidic acid interacts with ABI1 phosphatase 2C and regulates abscisic acid signaling". In: *Proceedings of the National Academy of Sciences of the United States of America* 101.25 (2004), pp. 9508–9513.

[2] Jian Zhao et al. "*Arabidopsis* phospholipase Dβ1 modulates defense responses to bacterial and fungal pathogens". In: *New Phytologist* (2013).

[3] Francesco Pinosa et al. "*Arabidopsis* Phospholipase Dδ Is Involved in Basal Defense and Nonhost Resistance to Powdery Mildew Fungi". In: *Plant physiology* 163.2 (2013), pp. 896–906.

[4] Maoyin Li et al. "Patatin-related phospholipase pPLAIIIβ-induced changes in lipid metabolism alter cellulose content and cell elongation in Arabidopsis". In: *The Plant Cell* 23.3 (2011), pp. 1107–1123.

Table S2: Primers used in this study

Primer ID	Sequence (5' –>3')		Purpose
		Adapter	Cloning
PLDα1-pF	cacc*GGATCC*GGCTTCGCTTTTGGGTTTTCT	cacc & *Bam*HI	PLDα1 genomic sequence and promoter
PLDα1-F	caccATGGCGCAGCATCTGTTGCA	cacc	PLDα1 genomic sequence
PLDα1-R1	TTAGGTTGTAAGGATTGGAGGCA	no	PLDα1 genomic sequence without stop codon for C-terminal fusion with YFP
PLDα1-R2	GGTTGTAAGGATTGGAGGCAGGTA	no	PLDα1 genomic sequence with stop codon
PLDδ-F	cacc*GGATCC*ATGGCGGAGAAAGTATCGGA	cacc & *Bam*HI	PLDδ genomic sequence
PLDδ-R	GC*GAATTC*TTACGTGGTTAAAGTGTCAGGAAGA	*Eco*RI	PLDδ genomic sequence with stop codon
PLDδ-R2	CGTGGTTAAAGTGTCAGGAAGAGCCA	no	PLDδ genomic sequence without stop codon for C-terminal fusion with YFP
H-PLDδ-pF	cacc*AAGCTT*GTCTCAGCCCATACAGCTCA	cacc & *Hind*III	PLDδ promoter
S-PLDδ-pR	GT*ACTAGT*GGTTACAACAATTCAGGTGGAA	*Spe*I	PLDδ promoter
		Gene locus	Genotyping
PLDα1-RP	CAAGGCTGCAAAGTTTCTCTG	*PLDα1*	PLDα1-RP/LP pair detects the WT allele,
PLDα1-LP	ATTAAGTGCAGGGCATTGATG	At3g15730	RP/LBa1 detects T-DNA.
PLDδ-RP	TCCGTTTGACCAGATCCATAG	*PLDδ*	PLDδ-RP/LP pair detects the WT allele,
PLDδ-LP	TTGCGATTATTACCAACAGCC	At4g35790	RP/LBa1 detects T-DNA.
LBa1	GCCATCGCCCTGATAGACGGTT	-	Genotyping of Salk T-DNA lines.
EDS6	GTGGAAACCAAATTTGACATTAG	*EDS1*	Genotyping of *eds1-2*.
EDS4	GGCTTGTATTCATCTTCTATCC	At3g48090	WT allele: 1500 bp + 750 bp
105/E2	ACACAAGGGTGATGCGAGACA		Mut allele:1500 bp + 600 bp
pad4-1F	GCGATGCATCAGAAGAGCA	*PAD4*	Amplicons are subject to *Bsm* FI digestion.
pad4-1R	GCGTTGTGCTCGCGTATCT	At3g52430	WT allele: 260 bp +108 bp after digestion.
sid2-2_F5	TTCTTCATGCAGGGGAGGAG	*SID2*	F5/R5 pair amplifies 7328 bp from the WT allele,
sid2-2_F6	CAACCACCTGGTGCACCAGC	At1g74710	but 581 bp from the *sid2-2* mutant allele.
sid2-2_R5	AAGCAAAATGTTTGAGTCAGCA		F6/R5 pair amplifies 879 bp from the WT allele.
RPW8.1-F	ATGCCGATTGGTGAGCTTGCGATA	RPW8.1	*RPW8.1* transgene
RPW8.1-R	TCAAGCTCTTATTTTACTACAAGC		
RPW8.2-F	ATGATTGCTGAGGTTGCCGCA	RPW8.2	*RPW8.2* transgene
RPW8.2-R	TCAAGAATCATCACTGCAGAACGT		
		Gene locus	qRT-PCR
AtPR1-F	AGAGGCAACTGCAGACTCATACAC	At2g14610	AtPR1-F/R detects *PR1* gene transcripts
AtPR1-R	AGCCTTCTCGCTAACCCACAT		
AtPDF1.2-F	TGTTCTCTTTGCTGCTTTCGACGC	At5g44420	AtPDF1.2-F/R detects *PDF1.2* gene transcripts
AtPDF1.2-R	TGTGTGCTGGGAAGACATAGTTGC		
AtUBC9-F	CAGTGGAGTCCTGCTCTCACAA	At4g27960	AtUBC9-F/R detects *UBC9* gene transcripts
AtUBC9-R	CATCTGGGTTTGGATCCGTTA		
